# Dynamic Responses of Endosymbiotic Microbial Communities Within *Microcystis* Colonies in North American Lakes to Altered Nitrogen, Phosphorus, and Temperature Levels

**DOI:** 10.3389/fmicb.2021.781500

**Published:** 2022-02-10

**Authors:** Christopher J. Gobler, Jennifer G. Jankowiak

**Affiliations:** School of Marine and Atmospheric Sciences, Stony Brook University, Southampton, NY, United States

**Keywords:** *Microcystis*, associated bacteria, next-generation sequencing, microbiome, eutrophication, warming (heating), phycosphere microorganisms

## Abstract

The toxic cyanobacterium, *Microcystis*, is a pervasive cyanobacterial harmful algal bloom (CHAB) - forming genus that naturally occurs in colonies that harbor diverse microbiomes of heterotrophic bacteria. While the effects of nutrient loading and climatic warming on CHABs are well-known, little is known regarding how these environmental drivers alter the structural and functional potential of the microbial assemblages associated with blooms that, in turn, may impact cyanobacterial growth. Here, we used next-generation sequencing of 16S ribosomal rRNA genes to characterize the dynamics of the bacterial assemblages within *Microcystis* colonies in two temperate North American lakes: Lake Erie and Lake Agawam (NY, United States) and quantified their responses to experimentally increased levels of nitrogen (N), phosphorus (P) and temperature. Across experiments, *Microcystis* populations were consistently and significantly promoted by N and, to a lesser extent, elevated temperature (*p* < 0.05). In contrast, bacterial assemblages within *Microcystis* colonies were more resilient to environmental perturbations, with the relative abundance of 7–16% of amplicon sequence variants changing and several individual taxa displaying significant (*p* < 0.05) increases and decreases in relative abundance, primarily in response to elevated temperature and to a lesser extent, N. In contrast to individual taxa, community diversity was not significantly altered by individual treatments during experiments but rather was inversely correlated with the intensity of *Microcystis* blooms (*p* < 0.001). While predicted metabolic function was even less impacted by environmental drivers than microbial diversity, the predicted abundance of nitrogenase (*nifH*), alkaline phosphatase (*phoX*), and urease (*ure*) genes significantly increased in response to N but decreased in response to increased temperature (*p* < 0.05). Collectively, the resilience of microbial community structure and function within colonies suggests they may support the ability of *Microcystis* to persist through short-term fluctuations in environmental conditions by supplying essential nutrients.

## Introduction

The global expansion of freshwater cyanobacterial blooms (CHABs) poses a significant threat to the sustainability of freshwater resources (Chorus and Bartram, 1999; Qin et al., 2010; Steffen et al., 2017; Huisman et al., 2018). Blooms of *Microcystis* are the most pervasive of the CHABs (Harke et al., 2016) and commonly produce the potent hepatotoxin microcystin that can be harmful to humans and animals (Stewart et al., 2008; Carmichael and Boyer, 2016). The intensification of CHABs in recent decades has been linked to accelerated cultural eutrophication and climatic change (Hudnell, 2008; Paerl and Huisman, 2008; O’Neil et al., 2012), and thus, environmental drivers have been a long withstanding focus of CHAB research (Carmichael, 2008; O’Neil et al., 2012; Visser et al., 2016). In recent years, however, the role of HAB-associated microbial symbionts, collectively termed the microbiome, in governing bloom formation and maintenance has gained attention (Seymour et al., 2017; Jackrel et al., 2019; Cook et al., 2020; Schmidt et al., 2020; Pound et al., 2021; Smith et al., 2021).

It is well-known that primary productivity in freshwater systems is largely regulated by the availability of the key limiting nutrients, phosphorus and nitrogen (Downing et al., 2001; Conley et al., 2009; O’Neil et al., 2012; Schindler, 2012), with nutrient excess often leading to increased cyanobacterial biomass (Harke et al., 2015). Rising surface water temperatures can further compound eutrophication by enhancing shifts in phytoplankton composition (Deng et al., 2014; Jankowiak et al., 2019) toward cyanobacterial-dominated communities (Paerl and Huisman, 2008; Paerl et al., 2011) as many cyanobacteria exhibit higher thermal optima than co-occurring algal species (Reynolds, 1984). While *Microcystis* blooms are promoted by temperature and nutrient enrichment, they have also been shown to persist under unfavorable conditions, such as periods of nutrient depletion following the rapid drawdown of nutrients by phytoplankton growth at the onset of blooms (Sarnelle, 1992; Heisler et al., 2008). Moreover, peak *Microcystis* abundances often coincide with warm water temperatures with low dissolved inorganic nitrogen concentrations (Sarnelle, 1992; Gobler et al., 2007; Stumpf et al., 2012). Microbial symbionts have been shown to help facilitate the survival of their hosts under a broader range of conditions by contributing toward essential functions (Lau and Lennon, 2012), for example, increasing algal tolerance to temperature and salinity changes (Dittami et al., 2016; Lian et al., 2018). It has becoming increasingly evident that microbiomes are key components to most living organisms and that the collective functions provided by these closely associated bacteria are intimately linked to the fitness and adaptability of their host species (David et al., 2019; Wilkins et al., 2019; Jackrel et al., 2020). Thus, the *Microcystis*-associated microbiome may play an integral role in the ability of *Microcystis* blooms to persist through optimal and sub-optimal conditions.

*Microcystis* has long been known to harbor diverse epiphytic and embedded bacteria within the mucilage that binds its colonial structure (Carr and Whitton, 1973; Worm and Søndergaard, 1998; Kim et al., 2019). Advances in molecular and computational technologies have allowed for a more detailed examination of these highly complex and dynamic algal-bacterial networks (Seymour et al., 2017; Liu et al., 2019; Chun et al., 2020; Jankowiak and Gobler, 2020). Many interactions are involved in resource allocation with the region surrounding algal cells termed the phycosphere (Bell and Mitchell, 1972) being a dynamic interface of intense bacterially mediated nutrient cycling and algal-bacterial metabolic exchange. Phytoplankton aggregates provide localized hotspots of organic matter (fixed carbon) consumed by bacteria while bacteria may provide access to newly assimilated and recycled nutrients, vitamins, and/or micronutrients (Seymour et al., 2017), perhaps to offsetting host nutrient deficits (Li et al., 2018). Beyond nutrition, the *Microcystis* colony also provides a favorable microenvironment for growth, providing a surface for bacteria to colonize within the water column with O_2_, organic carbon, and pH conditions conducive for bacterially mediated processes (Berry et al., 2017; Jankowiak and Gobler, 2020) while bacteria may help detoxify harmful compounds (i.e., ROS) and synthesize growth factors (i.e., auxins) which enhance phytoplankton productivity (Morris et al., 2011; Seymour et al., 2017; Cirri and Pohnert, 2019). As examples, xenic *Microcystis* cultures exhibit increased growth and productivity compared to axenic cultures (Kim et al., 2019; Jackrel et al., 2020) and microbiomes of *Microcystis* have been shown to provide a greater interspecific competitiveness (Schmidt et al., 2020; Hoke et al., 2021).

The complementary nature of the *Microcystis*-microbiome biochemical pathways has been proposed to stem from adaptive gene loss in *Microcystis*, in which genetic streamlining of metabolically intensive pathways occurs when the service provided can be readily obtained from the co-occurring community (Rocap et al., 2003; Morris et al., 2012). Genome reduction provides a selective advantage by reducing energy expended toward resource acquisition and lowering reproductive costs (Morris et al., 2012; Kazamia et al., 2016). There is increasing evidence of species- (Grossart et al., 2005; Sison-Mangus et al., 2014; Hattenrath-Lehmann and Gobler, 2017; Jackrel et al., 2020) and even genotype-specific (Jackrel et al., 2019; Kim et al., 2019; Smith et al., 2021) selective associations between HAB species and their symbiotic bacteria, supporting an interdependent relationship. Comparative metagenomic studies have revealed that the *Microcystis*-associated microbiome not only has a distinct and more highly conserved composition than co-occurring free-living bacteria across geographically (local, regional, and global) and temporally (seasonal) distinct blooms (Cai et al., 2014; Cook et al., 2020; Jankowiak and Gobler, 2020; Smith et al., 2021) but also forms more tightly coupled networks with a higher degree of positive interactions (Liu et al., 2019). These findings suggest bacteria with favorable functions may be maintained within *Microcystis* colonies, increasing *Microcystis*’ robustness to environmental change.

Although *Microcystis*’ response to environmental perturbations has been extensively researched, little is known about how the composition and subsequent functional potential of its associated microbiome is altered by these drivers. Bacterial colonization may be directly shaped by physical conditions (i.e., temperature regulation of metabolic activity) or through host-mediated chemical signaling mechanisms (i.e., chemotaxis, bacterial quorum sensing mimicking; Behringer et al., 2018; Wilkins et al., 2019). While these mechanisms may lead to a more conserved microbiome, they may also deter mutualistic symbionts whose relationship becomes deleterious under periods of environmental stress (i.e., sequestering resources from host under nutrient limitation) or recruit bacterial strains that provide similar functions but are differentially adapted to environmental conditions (Seyedsayamdost et al., 2011; Seymour et al., 2017; Cirri and Pohnert, 2019; Wilkins et al., 2019; Hoke et al., 2021). Such activities help maintain essential functions in the microbiome while increasing the phenotypic plasticity of the host (Jackrel et al., 2019; Wilkins et al., 2019) and have been evidenced by the functional convergence despite taxonomic divergence of the *Microcystis* microbiome across trophic gradients, ecosystems, and time (Crump et al., 2007; Jackrel et al., 2019; Cook et al., 2020; Jankowiak and Gobler, 2020; Smith et al., 2021). While some field studies of the bacterial communities within *Microcystis* colonies have found temporal variance strongly correlated with temperature (Jankowiak and Gobler, 2020), it is difficult to parse the impact of single variables in an environmental setting. The direct effects of temperature and nutrient availability on the growth of individual bacteria strains have been well-described in the literature (Rivkin et al., 1996; Pomeroy and Wiebe, 2001; Del Giorgio et al., 2008), but few studies have examined their direct impact on HAB-associated microbial communities. Moreover, the few studies that have explored this have been culture-based or examined the entire naturally occurring bacterial community rather than those within the phycosphere (Eigemann et al., 2013; Jackrel et al., 2020; Matson et al., 2020; Hoke et al., 2021). For example, experimental studies by Dziallas and Grossart (2011) identified a strong temperature dependency for the presence of select bacterial groups within the *Microcystis* microbiome, but such culture-based studies likely contain bottlenecked microbiomes (Kim et al., 2019). As the composition of a microbiome influences its functional potential and, in turn, can impact the fitness of the host (Jackrel et al., 2020; Jankowiak and Gobler, 2020), there is a need to understand how environmental factors impact complex, naturally occurring *Microcystis*-associated microbiomes, to better inform CHAB mitigation strategies under current and future predicted conditions.

To address this knowledge gap, here, we investigated the direct impact of nutrient enrichment and temperature elevation on the dynamics of naturally occurring *Microcystis* microbiomes using a combined colony isolation, incubation experiment, next-generation amplicon sequencing (16S rRNA), and predictive metagenome approaches. Specifically, nutrient (N and P) and temperature amendment experiments were conducted with bloom water from two temperate North American lakes, Lake Agawam (NY) and Lake Erie (Great Lakes) with the aim to: (1) Identify how these drivers alter composition and diversity of the bacterial assemblages embedded within *Microcystis* colonies, and (2) Assess how these drivers impact the genetic functional potential of the microbiomes, with a particular interest in conserved patterns of enrichment or depletion of functional groups (i.e., N and P assimilating bacteria) that may provide *Microcystis* with essential nutrients under limiting conditions, and (3) Explore correlations between bacterial community shifts and *Microcystis* abundance. Experiments were performed on bacterial communities within *Microcystis* colonies associated with a range of initial cyanobacterial densities and environmental conditions, and their responses were compared within and between lakes, to identify conserved patterns of change across the *Microcystis* microbiomes.

## Materials and Methods

### Study Sites

**Figure 1:**
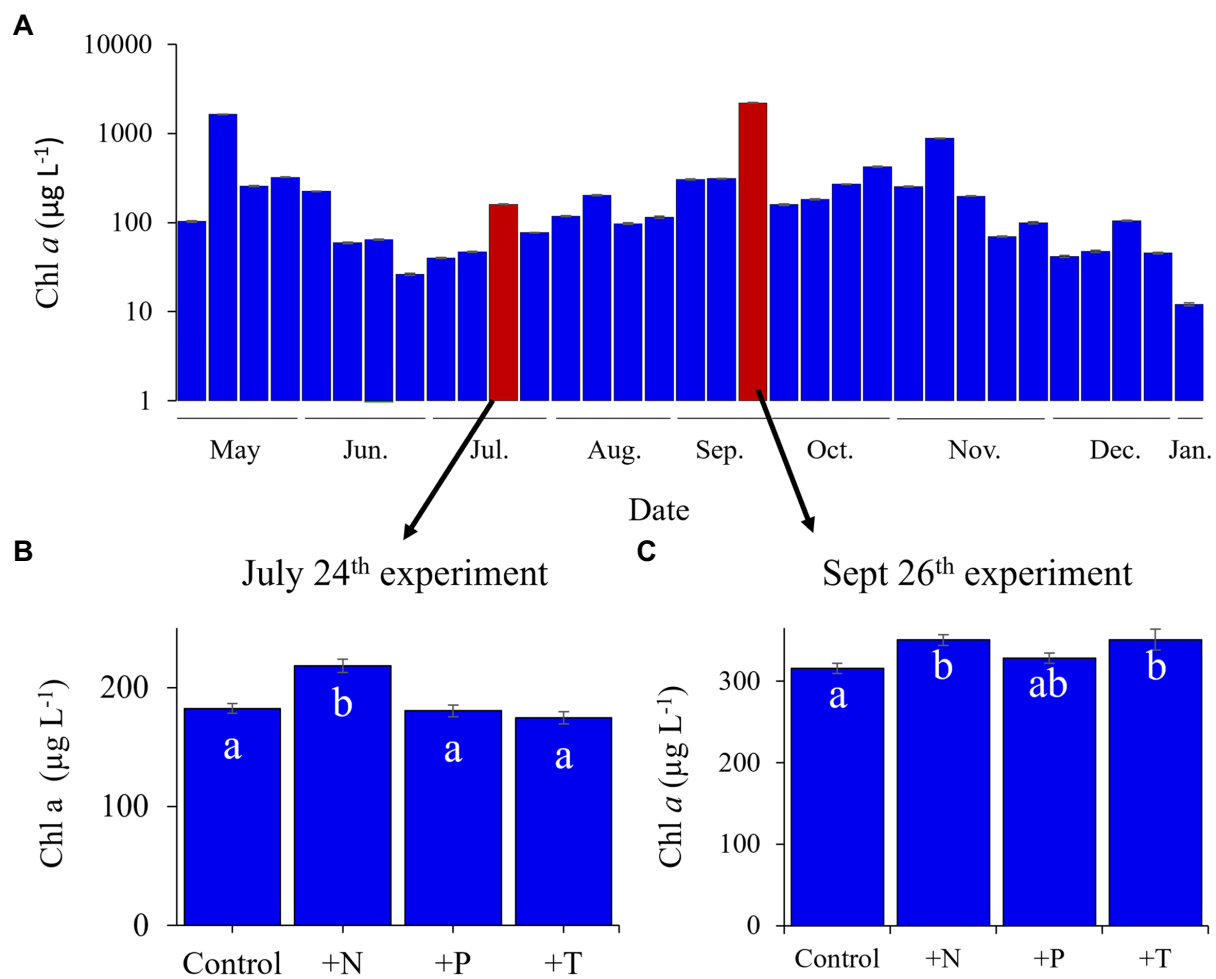
Lake Agawam phytoplankton community dynamics in 2017 determined *via* fluoroprobe. **(A)** Cyanobacterial dynamics throughout the monitoring period with highlighted bars representing experimental dates. Fluorometrically derived phytoplankton abundances in response to the experimental treatments (CTR, Control; +N, Nitrate addition; +P, Orthophosphate addition; +T, elevated incubation temperature) in the **(B)** July 25th and **(C)** the September 26th experiments. Letters indicate significant differences in cyanobacteria abundances between treatments, bars with shared letters are not significantly different. Note the September 26th experiment was conducted with lower density bloom water than the monitoring sample (collected south of the monitoring site).

**Figure 2:**
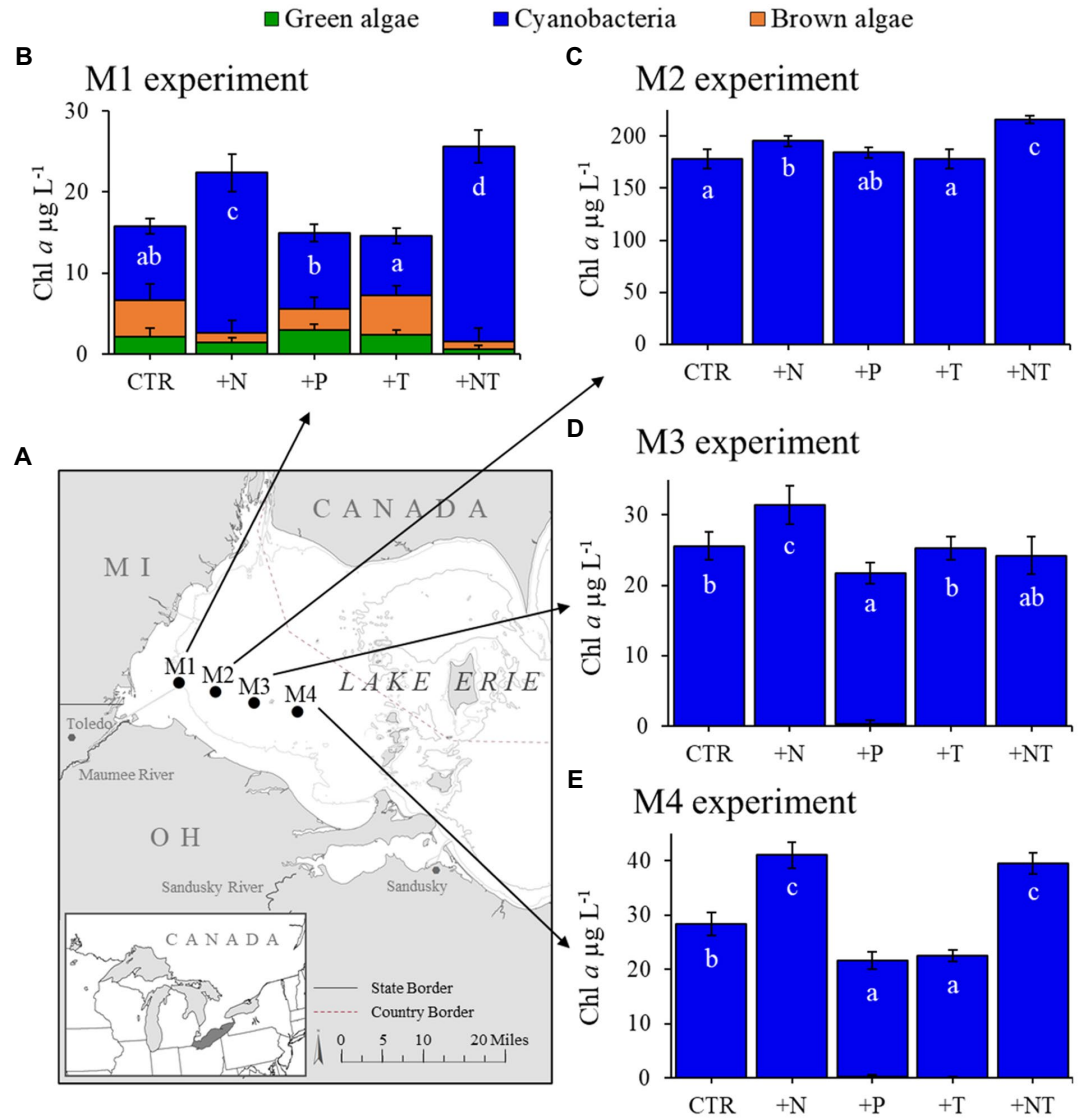
**(A)** Sampling locations of for experiments in Lake Erie’s western basin. **(B–E)** Response of the fluoroprobe-derived algal groups to experimental treatments (CTR, Control; +N, Nitrate addition; +P, Orthophosphate addition; +T, elevated incubation temperature). Letters indicate significant differences in cyanobacteria abundance between treatments, bars with shared letters are not significantly different.

Experiments were conducted with lake water from two temperate North American lakes, Lake Agawam and Lake Erie, during the summer of 2017. Both lakes are prone to annually recurrent *Microcystis*-dominated cyanobacterial blooms but are hydrodynamically distinct, allowing for the comparison of *Microcystis* microbiomes across a broad range of conditions. Lake Agawam (LA; 40.88148, −72.39274) is a small and shallow costal lake located on Long Island, NY (United States), which annually experiences dense, extended (six-month) *Microcystis* blooms (Gobler et al., 2007; Davis et al., 2009; Jankowiak and Gobler, 2020). In contrast, Lake Erie (LE; United States) is part of the socio-economically important Laurentian Great Lakes, which constitutes one of the largest freshwater systems in the world (Fuller et al., 2002; Gronewold et al., 2013). Lake Erie is the smallest (by volume; Davis et al., 2012), warmest (Stumpf et al., 2012) and most eutrophic (Mortimer, 1987; Steffen et al., 2014) of the Great Lakes, experiencing intensifying late summer *Microcystis* blooms in its western basin since the 1990s (Michalak et al., 2013; Steffen et al., 2014; Ho et al., 2017). In 2017, a *Microcystis* bloom was present in Lake Agawam from the beginning of monitoring in May through January 2018. Experiments (*details below*) were conducted during summer and fall on July 24th and September 26th (Figure 1). In Lake Erie, a severe *Microcystis* bloom developed in the western Basin in late July of 2017 and persisted through late October, peaking in August and again in mid-September (National Oceanic and Atmospheric Administration, 2017). Spatial transects of the bloom were conducted on September 18th and September 21st aboard the *R/V* Erie Monitor (The Ohio State University) that sampled four sites from the mouth of the Maumee River (M1) toward the Bass Islands in the east (M4; Figure 2). Experiments (*details below*) were conducted with water from sites M2 and M4 on September 18th and from M1 and M3 on September 21st (Figure 2). The experimental dates and sites were selected to capture a range of cyanobacteria densities that were confirmed on-site with a BBE Moldaenke Fluoroprobe (*see below*; Beutler et al., 2002; Chaffin et al., 2013; Harke et al., 2015).

### Nutrient-Temperature Amendment Experiments

Experiments were designed to assess the effects of elevated N, P, and temperature on the abundance of *Microcystis*, as well as the composition and functional potential of the colony-associated bacteria. For the Lake Agawam experiments, 60 L of subsurface lake water (~0.25 m) was collected in acid-washed 20 L carboys and transported to Stony Brook University Southampton for processing. The surface water was mixed and laminarly transferred into 12, acid-washed 4-L polycarbonate bottles. Triplicate bottles were then amended with one of the following treatments: 25 μm N (+N, as NH_4_Cl), 1.5 μm P (+P, as K_2_HPO_4_), +4°C above ambient lake temperature (+T), or left un-amended as a control. The nutrient additions were representative of pulses of nitrogen and orthophosphate that have been previously described in bloom prone systems (Wilhelm et al., 2003; Davis et al., 2015; Harke et al., 2015) while the 4°C increase is consistent with temperature projections for the 21st century (Houghton et al., 2001) and is representative of summer heatwaves within temperate latitudes (Joehnk et al., 2008). The bottles were incubated for 48 h in outdoor, temperature-controlled sea-tables covered with one layer of neutral density screening that reduced light by 33% to mimic *in situ* bloom conditions. The control and nutrient amended bottles were incubated at the ambient lake temperature, measured at the time of sample collection with a handheld YSI sonde (model 556), to mimic natural conditions while the +T treatment bottles were incubated in a second sea-table containing heating wands (ViaAqua Inc.) throughout to achieve the +4°C conditions. Temperatures/light conditions during incubations were measured continuously using *in situ* HOBO loggers (Onset Computer Corporation, MA, United States). Bottles were mixed daily to promote equal phytoplankton-nutrient distribution and were inspected for floating cyanobacterial colonies prior to mixing to confirm the cyanobacterial cells were still healthy within the bottle enclosures.

Parallel experiments were conducted for the Lake Erie sites with minor modifications. For each, experiment 60 L of subsurface lake water (~0.25 m) was collected in acid-washed 20 L carboys and transported to the Franz Theodore Stone Laboratory (The Ohio State University) for processing. Triplicate bottles were amended as above with the addition of a +NT treatment (+25 μm NH_4_Cl incubated at +4°C ambient lake temperature) to examine the interactive effects of nitrogen and temperature elevation. The bottles were incubated for 48 h in clear, submersible containers at 0.25 m depth in Fishery Bay, Lake Erie with screening as described above at either the ambient lake temperature, achieved with openings in the container allowing for lake water exchange, or an elevated temperature (+4°C) achieved with heating wands (ViaAqua Inc.) within the container. Incubations otherwise proceeded as described above.

At the initial and 48-h timepoints triplicate DNA samples were collected from each bottle to examine the composition of the *Microcystis* colony-associated bacteria (*see Heterotrophic bacteria fractionation for details*). Phytoplankton communities in each bottle were fluorometrically assessed with a BBE Moldaenke Fluoroprobe to estimate chlorophyll *a* (Chl *a*) abundance of the cyanobacteria, green algae, brown algae (e.g., diatoms, dinoflagellates, raphidophytes, and haptophytes) based on differential fluorescence of photosynthetic accessory pigments (Beutler et al., 2002; Chaffin et al., 2013; Harke et al., 2015). The signal of each channel was previously affirmed with >50 cultures of diatoms, cyanobacteria, dinoflagellates, raphidophytes, green algae, and haptophytes, and the cryptophyte levels were excluded from this study due to significant cross over of the cyanobacteria signal into the cryptophyte channel (Jankowiak et al., 2019). Additionally, duplicate samples for analysis of total (whole water) and dissolved (filtered through a combusted EMD Millipore APFB glass fiber filter) nutrients were collected from each bottle and stored at −20°C until further processing. Nutrient samples were analyzed for nitrate, ammonium, orthophosphate, total nitrogen (TN), and total phosphorus (TP) on a Lachat Instruments autosampler (ASX-520 series) using standard wet chemistry (Valderrama, 1981; Jones, 1984; Parsons, 2013) with 95 ± 10% recovery of standard reference material (SPEX CertiPrep^™^).

### *Microcystis* Colony Isolation and DNA Sequencing

To examine the heterotrophic bacteria closely associated with *Microcystis* colonies (epiphytic or embedded), the *Microcystis* colonies were isolated from the bloom water using a modified approach from Kapustina (2006) and Cai et al. (2013) and described in Jankowiak and Gobler (2020). Specifically, each bottle was well-mixed prior to filtering 1–2 L of water, dependent on the cyanobacterial density, through a 20-μm nylon mesh sieve to capture the *Microcystis* colonies while allowing free-living bacteria to pass into the filtrate. A 20-μm sieve was chosen based on the operational definition of a *Microcystis* colony provided by Worm and Søndergaard (1998). To remove large (>20-μm) non-*Microcystis* particles that were also captured by the filter, the biomass was then resuspended in 200 ml of bacteria-free, 0.2-μm filtered lake water. After a short period (minutes), the *Microcystis* colonies rose to the surface due to their high buoyancy while the non-*Microcystis* particles dropped out of suspension. The colonies were skimmed off the surface using a 50 ml serological pipet and resuspended twice more, as described, to further isolate colonies from non-colony particles. An aliquot of this fraction was then preserved with Lugol’s iodine solution (5% v/v) and examined *via* microscopy to confirm there was no contamination of other large particles or phytoplankton. Triplicate samples (50 ml) of the purified colonies, per bottle, were filtered onto 0.22 μm polycarbonate filters and immediately stored at −80°C until DNA extraction.

For molecular analyses, double-stranded DNA was extracted from the filters using the DNeasy PowerWater Kit (Qiagen; Venlo, Netherlands) per the manufacturer’s instructions. The resulting extracts were assessed for quantity and quality on a Qubit^®^ 4 fluorometer (Thermo Fisher) and normalized prior to sequencing at Molecular Research Laboratories (Shallowater, TX, United States) following the method described in Jankowiak et al. (2019). Specifically, the V4 region of the 16S SSU rRNA gene (~252 bp) was amplified using the universal primer set 515F: 5′-GTGYCAGCMGCCGCGGTAA-3′ (Parada et al., 2016) and 806R: 5′-GGACTACNVGGGTWTCTAAT-3′ (Apprill et al., 2015) to target the prokaryotic assemblages, sequenced on an Illumina MiSeq (2 × 300 bp) sequencer. The resulting reads were processed using QIIME 2 software (v2018.6; Bolyen et al., 2018) as described in Jankowiak and Gobler (2020). Briefly, the joined paired end reads, trimmed of their identification barcodes and primers using the Cutadapt plugin (Martin, 2011), were de-multiplexed into their respective samples using the DEMUX plugin and dereplicated into 100% amplicon sequence variants (ASVs) using the DADA2 plugin (Callahan et al., 2016). This is, to the best of our knowledge, one of the first studies to use this high level of stringency regarding identification of microbes associated with *Microcystis* blooms as previous studies have traditionally clustered reads into 97% similarity operational taxonomic units (OTUs; Cai et al., 2014; Louati et al., 2015; Akins et al., 2018; Cook et al., 2020). The ASV representative sequences were assigned taxonomies in QIIME 2 using a classifier trained with the SILVA rRNA (16S SSU) release v132 reference database (Quast et al., 2012) and confirmed using NCBI BLAST (Altschul et al., 1990). All mitochondria and chloroplast annotated features were then removed, since the focus of the study was on the prokaryotic assemblages associated with *Microcystis*, and the prokaryotic reads were split into cyanobacterial and heterotrophic bacterial data sets for statistical analysis. The raw sequence reads from this study have been deposited to National Center for Biotechnology Information SRA database under deposited to NCBI SRA database (SRA bioproject PRJNA601166, Accession: SRX7554361-SRX7554236).

### Statistical Analyses

To test for effects of the experimental treatments (+N, +P, +T, +NT) on cyanobacteria biomass, three-way ANOVAs were conducted on the fluoroprobe-derived cyanobacterial abundances in R statistical environment v 3.6.2 (R Development Core Team, 2013). Prior to analysis, data were assessed for normality (Shapiro-Wilk test) and equal variance (Bartlett’s test) and then modeled with a linear model (Gaussian error structure) checked for goodness of fit through examination of qqplots and a histogram of the residuals. Left skewed data sets were square root transformed to increase normality. Three-way ANOVAs were then performed on the models using the car package in R to determine significant main effects and interactions of N, P, and T, followed by a Tukey *post hoc* multiple comparison analysis between treatments.

All statistical analyses on 16S rRNA sequencing data were performed in QIIME 2 v2018.6 (Bolyen et al., 2018) unless otherwise noted. To investigate the effects of the nutrient and temperature treatments on the structure of the *Microcystis*-associated microbiomes, ASV-inferred alpha (observed ASVs, Shannon richness, and Pielou’s evenness) and beta (Bray-Curtis dissimilarity) diversity metrics were calculated for the bacterial ASV data set (cyanobacterial reads removed). Prior to analysis, the data set was rarefied to a sampling depth of 13,734 reads (the smallest library size), using the QIIME 2 core metrics pipeline. Significant differences in the alpha diversity metrics between treatments and experiments were assessed using Kruskal-Wallis pairwise tests with Benjamini and Hochberg multiple comparison correction (Kruskal and Wallis, 1952). To visualize significant differences in the bacterial community structures (beta diversity) between the experiments and treatments principal coordinates analyses (PCoA) were conducted on the ASV-inferred Bray-Curtis dissimilarities, followed by permutational multivariate analysis of variance analysis (PERMANOVA; 999 permutations calculated per test) to identify significantly different groups. To determine whether changes in the microbiome communities impacted *Microcystis* growth, significant correlations between the bacterial alpha and beta diversities and the biological parameters (Fluoroprobe-derived cyanobacteria abundance) were assessed using Mantel tests (999 permutations) and Spearman correlations, respectively.

To assess changes in the abundance of individual bacterial taxa between treatments a differential abundance analysis was conducted on each experiment using the Phyloseq and DESeq packages (Anders and Huber, 2010; McMurdie and Holmes, 2013, 2014) in R v 3.6.2 (R Development Core Team, 2013). Briefly, the raw 16S rRNA read abundances were normalized with the median ration method prior to modeling using a negative binomial distribution with parametric fitting of the dispersions. Significant log_2_ fold changes in abundance (*α* = 0.05) were then determined with Wald significance testing and the resulting values of *p* were adjusted to correct for multiple testing using the Benjamini-Hochberg procedure. Significant log_2_ fold changes in abundances were calculated on individual taxa grouped at all taxonomic levels (phylum through ASV) to account for variations in depth of taxonomic identification. Shared and unique taxa within treatments were identified analysis with Venny software v2.1 (Oliveros, 2007).

To investigate how the potential functional capabilities of the *Microcystis*-associated microbiomes were altered by the experimental treatments Phylogenetic Investigation of Communities by Reconstruction of Unobserved States (PICRUSt) software (Langille et al., 2013; Louca and Doebeli, 2017; Czech and Stamatakis, 2018; Kozlov et al., 2018) was used to create predicted composite metagenomes for each sample. PICRUSt uses a phylogenetic reconstruction algorithm with marker gene data, such as 16 s rRNA abundances, and the KEGG genome reference database to predict the abundance of gene families (KO; KEGG functional orthologs) within a community. Prior to analysis, the 16S rRNA ASV heterotrophic bacterial abundances were normalized by converting to relative abundances and the resulting KO-inferred predicted metagenomes were visualized with PCoA analysis using the QIIME2 diversity plugin to access similarities in the community functional potentials between treatments. Additionally, the differential abundance of predicted KOs were further investigated using STAMPS software v2.1.3 (Parks et al., 2014) and significant genes were displayed with heatmaps created using Morpheus software^1^ to identify patterns of differential abundance among gene families of interest (i.e., N and P cycling) between treatments. To validate the metagenomic predictions made *via* PICRUSt analyses, the total relative abundance of taxa with known sequences of the nitrogen fixation *nif*H and alkaline phosphatase *pho*X genes in the NCBI nucleotide database were manually compared among the treatments. Specifically, all *nifH* or *pho*X sequences classified as heterotrophic bacteria in the NCBI nucleotide database and their associated taxonomies were downloaded as a reference database. The database was then used to filter the QIIME ASV frequency table based on taxonomic assignment to extract abundances of all potentially *nif*H or *pho*X-containing taxa and the cumulative relative abundance was analyzed with ANOVA and Tukey *post hoc* analysis in R.

## Results

### *In situ* Cyanobacterial Bloom Characteristics and Growth Response

During 2017, a dense cyanobacterial bloom was present in Lake Agawam from the start of sampling on May 8th (103 μg cyanobacterial pigments L^−1^ as quantified fluorometrically) through the end of December (45.6 μg L^−1^, Dec 26th; Figure 1A). During this eight-month bloom, levels peaked in the spring and late summer at 1,637 μg L^−1^ on May 17th and 2,209 μg L^−1^ on September 26th (Figure 1A). *Microcystis* was the dominant cyanobacteria by biomass in all monitoring samples as determined *via* microscopic examination (Jankowiak and Gobler, 2020). The Lake Agawam experiments on July 24th and Sept 26th exhibited intermediate (254 μg L^−1^) and high (370 μg L^−1^) initial cyanobacterial levels, respectively, with the Sept 26th sample collected from a site slightly separated from the routine monitoring sample in time and space. Relatively high *in situ* surface water temperatures (~24°C) and low nitrate (<1 μm) and orthophosphate levels below detection limit (0.05 μm) were present at the time of water collection for both experiments, however higher ammonium levels were present during the Sept vs. July experiment (~3.5 μm vs. ~1 μm; Table 1). In Lake Erie, on September 18th, cyanobacteria levels generally declined with increasing distance from the Maumee River, peaking at site M2 (195 μg L^−1^), while on September 21st cyanobacteria biomass was elevated to the east (sites M3 and M4), with highest level of 23.9 μg L^−1^ at site M3 (Table 1). Surface water temperatures were ~21°C at all sites, while nitrate concentrations ranged from 0.57 μm at site M1 to nearly 6 μm at site M3, and ammonium concentrations ranged from 0.12 μm at site M3 to 3.44 μm at site M2 (Table 1). Orthophosphate concentrations were below the detection limit at all sites (Table 1).

**Table 1:** Environmental conditions within Lake Erie and Lake Agawam during sample collection for each experiment, as well as the mean experimental temperatures.

Experiment	Lake	Collection date	Coordinates		Lake conditions							Average experiment temperature (°C)	
			North	West	Cyanobacteria (μg L^−1^)	Surface temperature (°C)	Nitrate (μm)	Ammonia (μm)	Phosphorus (μm)	TN (mg N L^−1^)	TP (μg P L^−1^)	Ambient	Elevated
M1	Lake Erie	21-09-2017	41.76783	−83.3259	12.8	21.2	0.57 ± 0.07	0.81 ± 0.2	BDL	NA	NA	23.5	26.07
M2	Lake Erie	18-09-2017	41.7533	−83.2388	192	20.6	3.7 ± 0.24	3.44 ± 1.11	BDL	327.85 ± 15.45	8.41 ± 0.45	21.65	24.2
M3	Lake Erie	21-09-2017	41.73598	−83.1472	23.9	20.8	5.78 ± 0.23	0.12 ± 0	BDL	NA	NA	23.5	26.07
M4	Lake Erie	18-09-2017	41.72192	−83.0439	27.3	20.5	1.7 ± 0.24	1.42 ± 0.13	BDL	60.43 ± 7.09	1.79 ± 0.12	21.65	24.2
July 24th	Lake Agawam	25-07-2017	40.88144	−72.3928	254	24.4	0.68 ± 0.2	0.68 ± 0.09	BDL	61.18 ± 4	2.53 ± 0.14	22.95	29.49
Sept 26th	Lake Agawam	26-09-2017	40.88144	−72.3928	370	24.2	0.09 ± 0	3.52 ± 0.23	BDL	336.58 ± 22.72	14.62 ± 1.11	21.79	29.81

TN and TP is total nitrogen and phosphorus.

Across all experiments, the +N treatments had the greatest impact on cyanobacterial growth, yielding significantly higher cyanobacterial levels compared to the control in all six experiments (*p* < 0.05 for all; Figures 1; 2; precise values of *p* listed in Table 2). The greatest increase occurred in the Lake Erie M1 experiment which had a mixed initial phytoplankton community and the lowest cyanobacteria levels, as well as low initial nitrate and ammonium concentrations (Figure 2B; Table 1). Temperature was the next most influential treatment, yielding significantly higher cyanobacterial levels in the September Lake Agawam experiment (*p* = 0.007; Figure 1C; Table 2), but significantly lower levels during the M4 experiment in Lake Erie (*p* = 0.0004; Figure 2E; Table 2). There was a significant interaction between N and T in all Lake Erie experiments (Table 2), with synergistic increase in cyanobacteria levels compared to the control during the M1, M2, and M4 experiments, but antagonistic interaction during the M3 experiment (Figure 2). The +P treatment caused a significant decline in cyanobacteria biomass in the M3 and M4 experiments (*p* = 0.023, *p* = 0.0001 respectively; Figure 2; Table 2) and never yielded a significant increase in cyanobacterial biomass.

**Table 2:** ANOVA and multiple comparison results testing the effects on experimental variables on densities of cyanobacteria.

Experiment	Assumptions		Main effect				Multiple comparison									
	Normality	Equal variance	Nitrogen	Phosphorus	Temperature	N:T	CTR-N	CTR-P	CTR-T	CTR-NT	N-NT	N-P	N-T	P-T	P-NT	T-NT
July 24th	0.195	0.985	**<0.001**	0.598	0.087		<0.001	0.957	0.281			<0.001	<0.001	0.505		
Sept 26th	0.448	0.549	**0.015**	0.437	**0.002**		0.007	0.416	0.007			0.067	1.000	0.062		
LEM1	0.378	0.841	**<0.001**	0.052	0.322	**<0.001**	<0.001	0.996	0.067	<0.001	0.007	<0.001	<0.001	0.039	<0.001	<0.001
LEM2	0.849	0.242	**<0.001**	0.201	**0.020**	**0.020**	0.049	0.760	1.000	<0.001	0.020	0.285	0.049	0.759	<0.001	<0.001
LEM3	0.312	0.576	**<0.001**	**0.002**	**0.001**	**0.001**	0.002	0.023	0.998	0.713	<0.001	<0.001	0.002	0.037	0.161	0.867
LEM4	0.886	0.301	**<0.001**	**<0.001**	**<0.001**	**0.005**	<0.001	<0.001	<0.001	<0.001	0.446	<0.001	<0.001	0.842	<0.001	<0.001

Bolded values are statistically significant main effects.

### Microbiome Sequencing

The 26 Lake Agawam and 64 Lake Erie samples collectively generated a total of 5,241,829 16S rRNA sequences after joining and quality filtering, of which 609,489 were identified as mitochondria or chloroplast and not considered for further analysis. The remaining 4,632,340 sequences clustered into 1,915 ASVs (100% similarity), of which 1,840 were classified as heterotrophic bacteria and 75 were classified as cyanobacteria. Among individual experiments the following number of unique bacterial/cyanobacterial ASVs were identified: July 24th: 673/20, September 26th: 544/20, M1: 625/32, M2: 387/16, M3: 767/35, and M4: 729/33.

### Isolation of the *Microcystis* Microbiome From the Bulk Bacterial Community

As previously reported, 16S rRNA sequencing revealed *Microcystis* was a predominant cyanobacteria in the isolated *Microcystis* colony fraction, accounting for 25–67% of the cyanobacterial reads in the initial communities (Supplementary Figure 1; Jankowiak and Gobler, 2020). *Microcystis* reads mapped primarily to a genotype identified as *Microcystis* PCC 7914. The small filamentous cyanobacterium *Pseudanabaena* (identified primarily as *Pseudanabaena* PCC-7429), which is often an epiphyte of *Microcystis* colonies (Berry et al., 2017), was also present in high abundances accounting for 32–74% of initial cyanobacterial reads (Supplementary Figure 1; Jankowiak and Gobler, 2020). Together these two cyanobacteria composed 90–99% of the initial cyanobacterial reads except for Lake Erie site M4 where they were 66% of the reads (Jankowiak and Gobler, 2020), indicating a successful separation of the *Microcystis* colonies across experiments. The filamentous cyanobacteria *Dolichospermum* (*Dolichospermum* NIES41) and *Aphanizomenon* (*Aphanizomenon* MDT14a and NIES81) contributed 20% and ~10% of the initial reads, respectively, in the Lake Erie M4 experiment (Supplementary Figure 1).

### Composition of the Heterotrophic Bacteria Microbiome Assemblages

**Figure 3:**
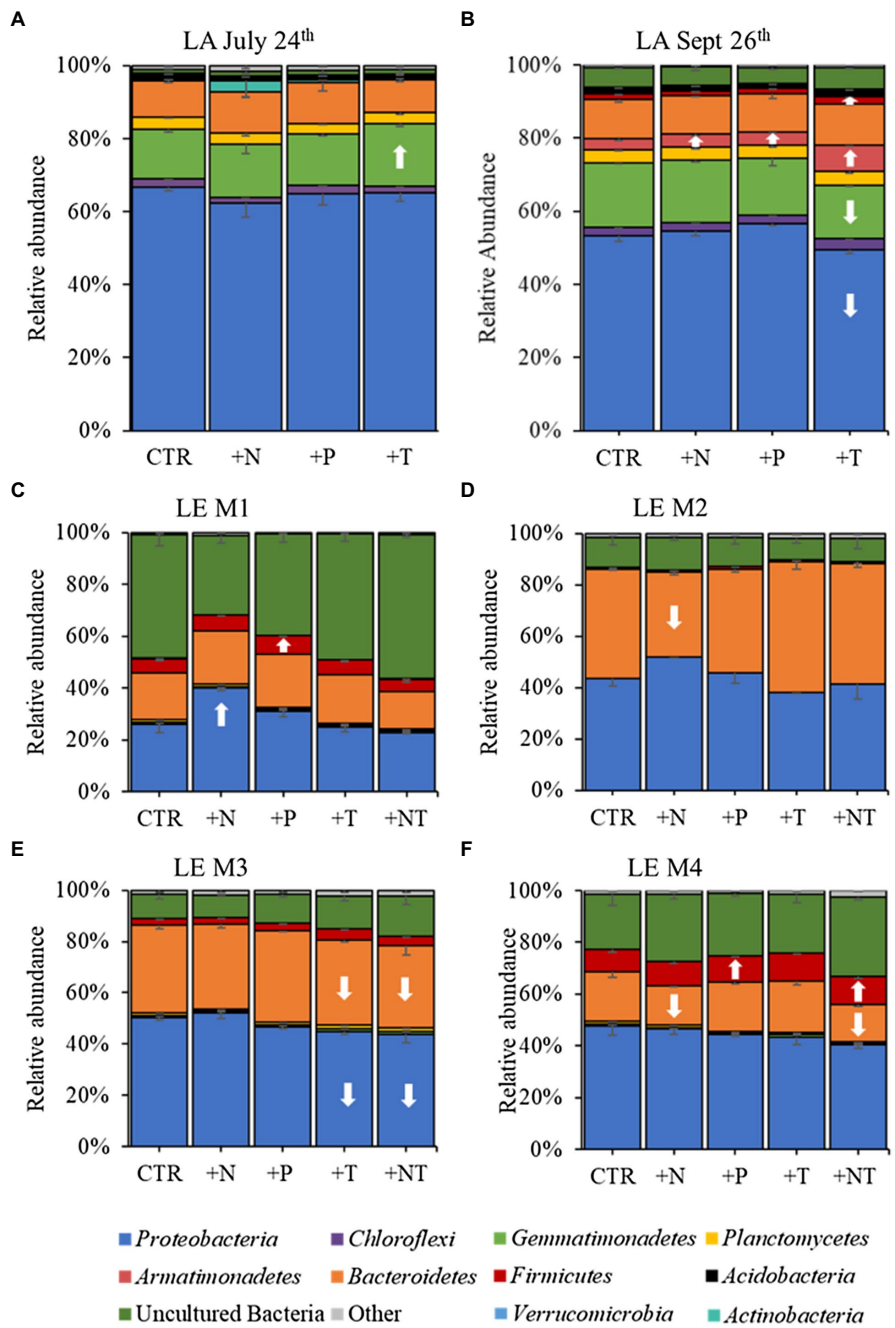
16S rRNA derived relative abundances of bacterial phyla in the **(A)** LA July 24th, **(B)** LA September 26th, **(C)** LE M1, **(D)** LE M2, **(E)** LE M3, and **(F)** LE M4 experiments. All low abundance phyla have been grouped into the “Other” category. Arrows indicate a significant increase (up) or decrease (down) of the respective phylum in each treatment (CTR, Control; +N, Nitrate addition; +P, Orthophosphate addition; +T, elevated incubation temperature) compared to the control.

16S rRNA sequencing of the heterotrophic bacteria associated with *Microcystis* colonies revealed similar patterns of bacterial groups across experiments (Figure 3). At the phylum level Proteobacteria and Bacteroidetes were dominant in all experiments, with Proteobacteria accounting for over half the heterotrophic bacterial reads in the Lake Agawam experiments (59.1 ± 6.49%) and slightly less than half in the Lake Erie experiments (42.4 ± 7.10), while Bacteroidetes (largely Cytophagales) accounted for 23.1 ± 12.6% of bacterial reads across all experiments (Figure 3). In Lake Agawam, the Proteobacteria phylum was composed primarily of Betaproteobacteria (~30–40% reads) followed by Alphaproteobacteria (~15% reads; Rhizobiales 3%) and Gammaproteobacteria (~5–10% reads; Supplementary Figure 2). In Lake Erie, the Alphaproteobacteria were most abundant among the Proteobacteria (~10–30% reads), followed by the Beta- (~10–15%) and Gammaproteobacteria (~5–10% reads; Supplementary Figure 2). In both lakes, the Betaproteobacteria were primarily composed of the Burkholderiaceae family and the *Gammaproteobacteria* of the Xanthomonadales order; the Alphaproteobacteria members differed between lakes, primarily belonging to the Rhizobiales order in Lake Agawam and the Caulobacterales, Acetobacterales, Rhodobacterales, Rickettsiales orders in Lake Erie. The remaining bacterial community composition was more variable between lakes. In Lake Agawam, the Gemmatimonadetes phylum accounted for 15.5 ± 1.62% bacterial reads in both experiments (Figure 3). Further, around 3% of the reads belonged to the Planctomycetes in both Lake Agawam experiments and the Armatimonadetes (largely Fimbriimonadales) composed 4.16 ± 1.94 in the Sept 26th experiment (Figure 3). In the Lake Erie experiments, a high percentage of reads were classified as uncultured bacteria, accounting for up to 55% of the heterotrophic bacteria reads in M1 (Figure 3). The Firmicute phylum accounted for, on average, 3–10% (largely Bacillales) of the Lake Erie bacterial reads except for M2 (Figure 3). All remaining phyla in both lakes were present in low abundances (<1% bacterial reads; Figure 3).

### Effects of Experimental Treatments on Bacterial Abundance

**Figure 4:**
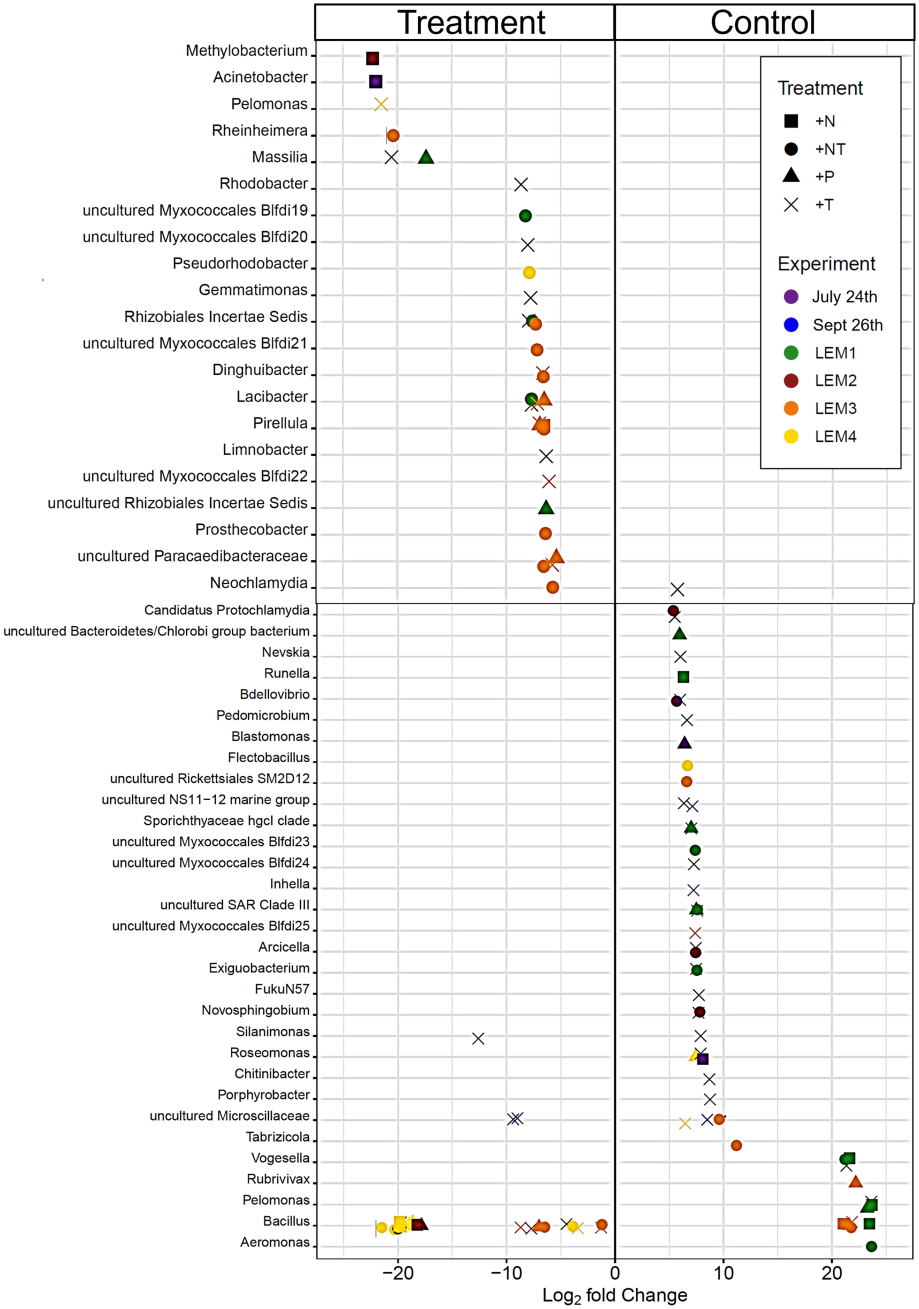
Bacterial ASV’s found to be significantly differentially abundant between the control and treatments among the experiments ordered by degree of fold change. Points with positive log fold changes are significantly enriched in the control, points with negative log fold changes are significantly enriched in the treatment. Only ASV’s with a log 2-fold change greater than 5 are shown.

**Figure 5:**
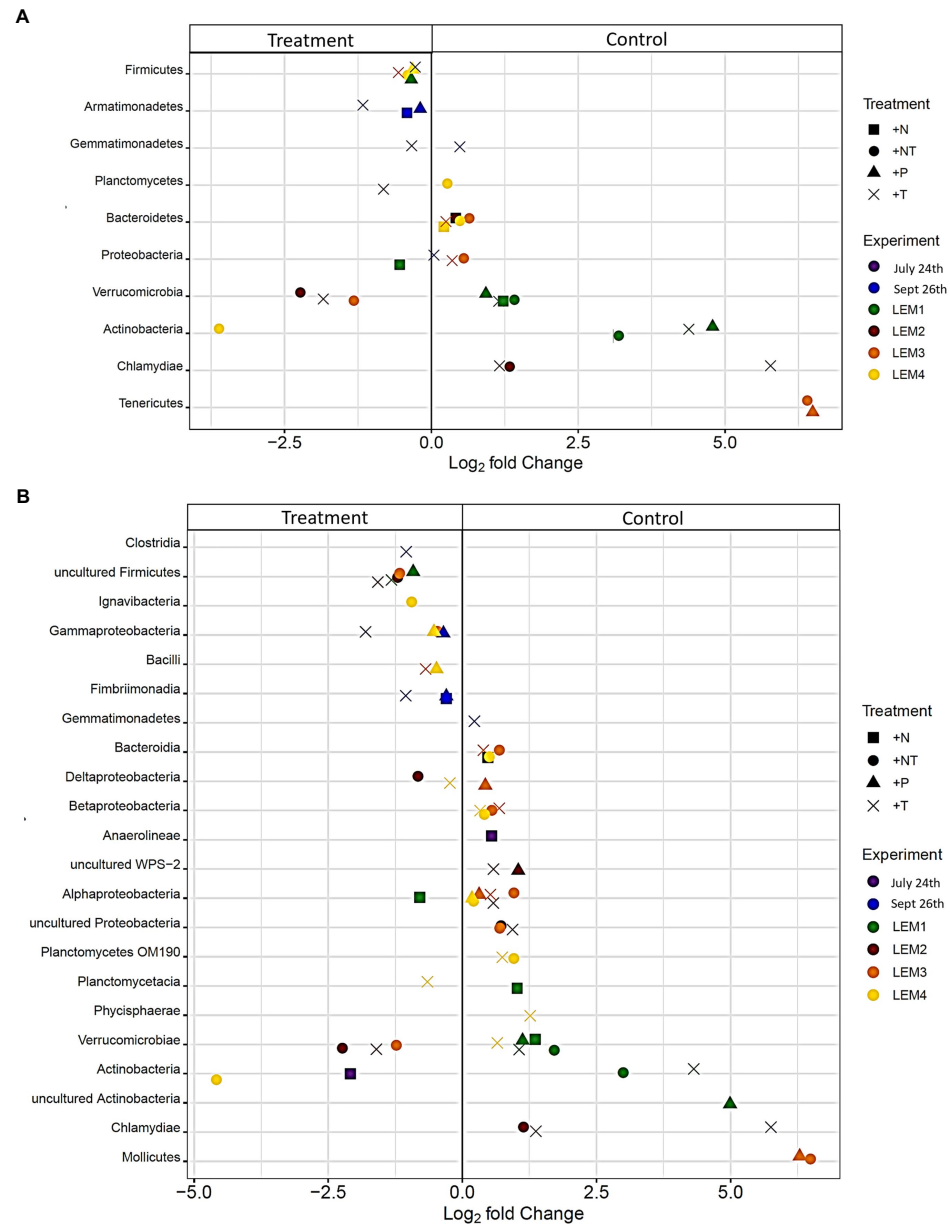
Bacterial taxa found to be significantly differentially abundant between the control and treatments among the experiments ordered by degree of fold change. Points with positive log fold changes are significantly enriched in the control, points with negative log fold changes are significantly enriched in the treatment with taxonomy divided by **(A)** phyla and **(B)** class.

**Figure 6:**
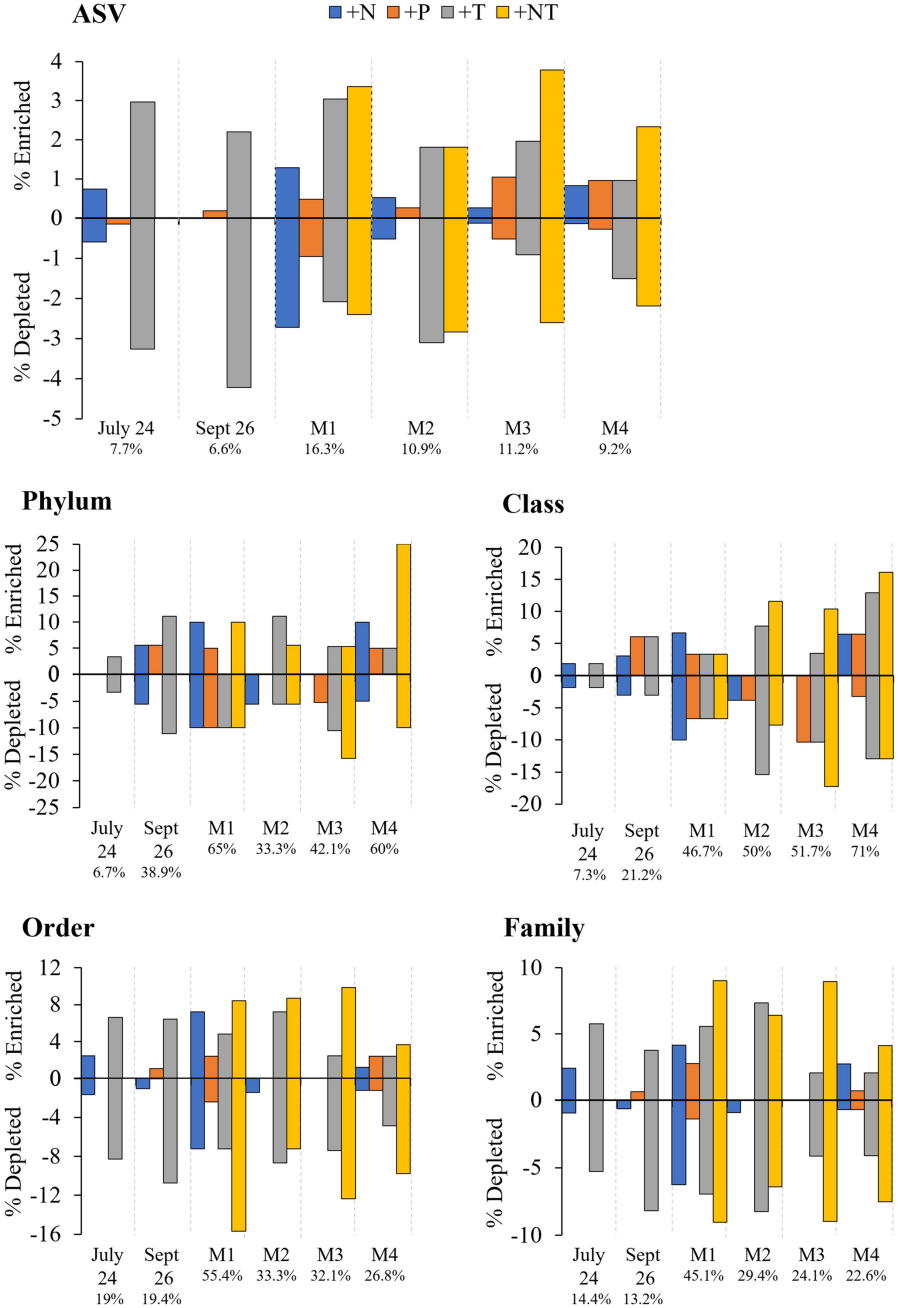
Percent enrichment and depletion of amplicon sequence variants (ASVs) per treatment. Number under experiment label indicates total percent ASVs altered across treatments per experiment.

In each experiment, around a third of the bacterial ASVs were present in the core microbiome, shared between the initial and control (37.7 ± 2.91%), and across all treatments (33.5 ± 3.53%; Supplementary Table 1). This increased to around 50–80% of taxa at higher taxonomic levels (Supplementary Table 1). Almost all unique ASVs/taxa were rare, accounting for <1% of the reads per sample, suggesting that community bottlenecking and bacterial exchange with the free-living community during bottle incubations were not significant. Differential abundance analysis, however, revealed experimental conditions did significantly alter the abundance of individual heterotrophic bacterial taxa during experiments (Figures 4–6; *p* < 0.05 for all; individual values of *p* listed in Supplementary Table 2).

In both Lake Agawam experiments, less than 10% of the ASVs were significantly differentially abundant between the treatments and the control (Figures 4, 6). Specifically, 51 of the 674 ASVs present in the LA July 24th experiment and 36 of the 544 ASVs present in the September 26th were specifically altered by a single treatment. The Proteobacteria phylum experienced a relatively low (<1 log_2_-fold change) but significant depletion in the +T treatment in the September 26th experiment (*p* = 0.048; Figure 3). Of the Proteobacterial classes, the Gammaproteobacteria was the only class to be significantly altered by the treatments, with a nearly 2 log_2_-fold enrichment in the July 24th +T treatment and a <1-log fold enrichment in the September 26th +P treatment (Supplementary Figure 2). The relative abundance of the Gemmatimonadetes phylum was significantly but differentially influenced by the +T treatment, being enriched in the July 24th experiment but depleted in the September 26th experiment (*p* = 0.047, 0.0065 respectively; Figure 3). The Armatimonadetes phylum was highly influenced by the treatments in the September 26th experiment, becoming significantly enriched in +N (*p* = 0.019), +P (*p* = 0.002), +T (*p* < 0.0001), compared to the control (*p* < 0.05; Figure 3).

Bacterial taxa in the Lake Erie M1 experiment were the most influenced by the treatments with 55 of the 625 ASVs significantly altered by one or more of the treatments (Figures 4, 6), among which the *Pelomonas* bacterium (Burkholderiales order) was the most altered experiencing an over 20 log_2_-fold depletion in all four treatments (Figure 4). Members of the Proteobacteria phylum (*p* = 0.00003) and Alphaproteobacteria sub-class (*p* < 0.0001) were both significantly enriched in the +N treatments compared to the control (Figure 3; Supplemental Figure 2), while the Firmicutes phylum was significantly enriched in the +P treatment (*p* = 0.0001; Figure 3). Notably, the rare Actinobacteria phylum (<1% bacterial reads) was highly significantly and over three log_2_-fold depleted in the +P (*p* < 0.00001), +T (*p* < 0.00001) and +NT (*p* = 0.00008) treatments compared to the control (Figure 5A).

In the other three Lake Erie experiments, around 10% bacteria were differentially abundant between treatments (Figure 6). In the M2 experiment, 25 of the 387 bacterial ASVs were significantly altered by one or more treatments (Figure 4). Notably, *Bacillus* (Firmicutes) was significantly enriched in all four treatments compared to the control by a nearly 20 log_2_-fold change (*p* = 0.000006, 0.00001, 2.39E-8, 2.06E-8 in +N, +P, +T, +NT respectively; Figure 4). Among the high abundance groups, the Alphaproteobacteria and the Bacteroidetes experienced relatively low (<1-log fold change) but significant changes in the relative abundance, being significantly depleted in the +T (*p* = 0.005) and +N (*p* = 0.005) treatments, respectively (Figures 3, 5; Supplementary Figure 2).

The relative abundance of 56 of the 767 ASVs present in M3 experiment were significantly altered by one or more treatments (Figures 4, 6), among which *Bacillus* (Firmicutes) was again highly influenced (>20 log_2_-fold change), but significantly depleted (+N: *p* = 0.002, +P: *p* = 0.0003, +T: *p* = 0.0002, +NT: *p* = 0.00006) in all four treatments compared to the control (Figure 4). Both the Proteobacteria and Bacteroidetes phyla were significantly depleted in the +T (*p* = 0.00004, 0.008 respectively) and +NT (*p* = 3.48E-7, 0.0004, respectively) treatments compared to the control, but with relatively low change in relative abundance (<1-log_2_-fold change) while members of the Firmicutes phylum were significantly enriched in the +T treatment (*p* = 0.008; Figure 3). Within the Proteobacteria, the Alphaproteobacteria and Betaproteobacteria were significantly depleted in the +T (*p* = 2.1E-6, 0.001) and +NT (*p* = 2.1E-13, 0.001) treatments, while the Gammaproteobacteria were significantly enriched in the +NT treatment (*p* = 0.002; Supplementary Figure 2). Further, the relative abundance of the Alphaproteobacteria was significantly depleted in the +P treatment (*p* = 0.006; Supplementary Figure 2). The Tenericutes phylum, while at low starting abundance (<1% bacterial reads; Figure 3) became more than six log_2_-fold depleted in the +P and +NT treatments (Figure 5A).

In the M4 Lake Erie experiment, the bacteria were the least influenced by the treatments, with only 43 of 729 ASVs significantly altered by one or more treatments (Figures 4, 6). Among the dominant phyla, there was a relatively small (<1 log_2_-fold) but significant (*p* < 0.05) change in the relative abundance of the Bacteroidetes and Firmicutes phyla, with the Bacteroidetes significantly depleted in both N addition treatments (+N: *p* = 0.036, +NT: p = 0.002) and the Firmicutes significantly enriched in the +P (*p* = 0.003) and +NT (p = 0.008) treatments (Figure 3). The Alphaproteobacterial and Betaproteobacterial classes were also significantly altered, both being depleted in the +NT treatment (*p* = 0.01, 0.006), as well as the +P and +T treatments, respectively, (*p* = 0.038, 0.004; Supplementary Figure 2). Among lower abundance phyla (<1% bacterial reads; Figure 3), the Actinobacteria were significantly enriched by over three log_2_-fold in the +NT treatment compared to the control (*p* = 0.006; Figures 3, 5A).

### Effects of Environmental Drivers on the Microbiome Community Structure

**Figure 7:**
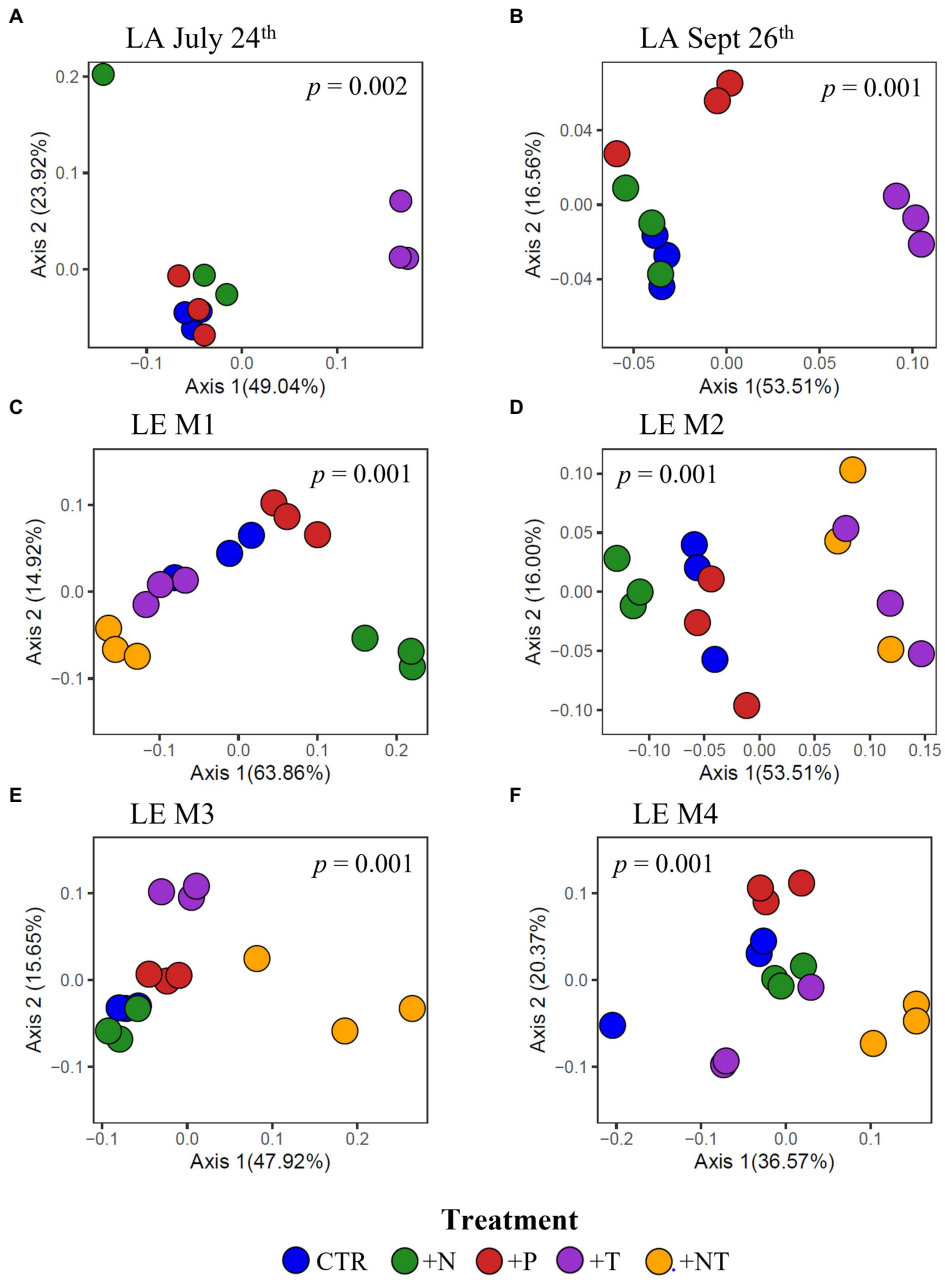
Principal coordinates analysis (PCoA) conducted on ASV abundances showing the dissimilarity of heterotrophic bacteria compositions between samples in the **(A)** LA July 24th, **(B)** LA September 26th, **(C)** LE M1, **(D)** LE M2, **(E)** LE M3 and **(F)** LE M4 experiments. Color denotes the sample treatment (CTR, Control; +N, Nitrate addition; +P, Orthophosphate addition; +T, elevated incubation temperature). Precents listed on the axes represents the percent of variation explained by PC1 and PC2. Value of *p* indicates the significance of the main effect of treatment on the communities determined *via* PERMANOVA.

**Figure 8:**
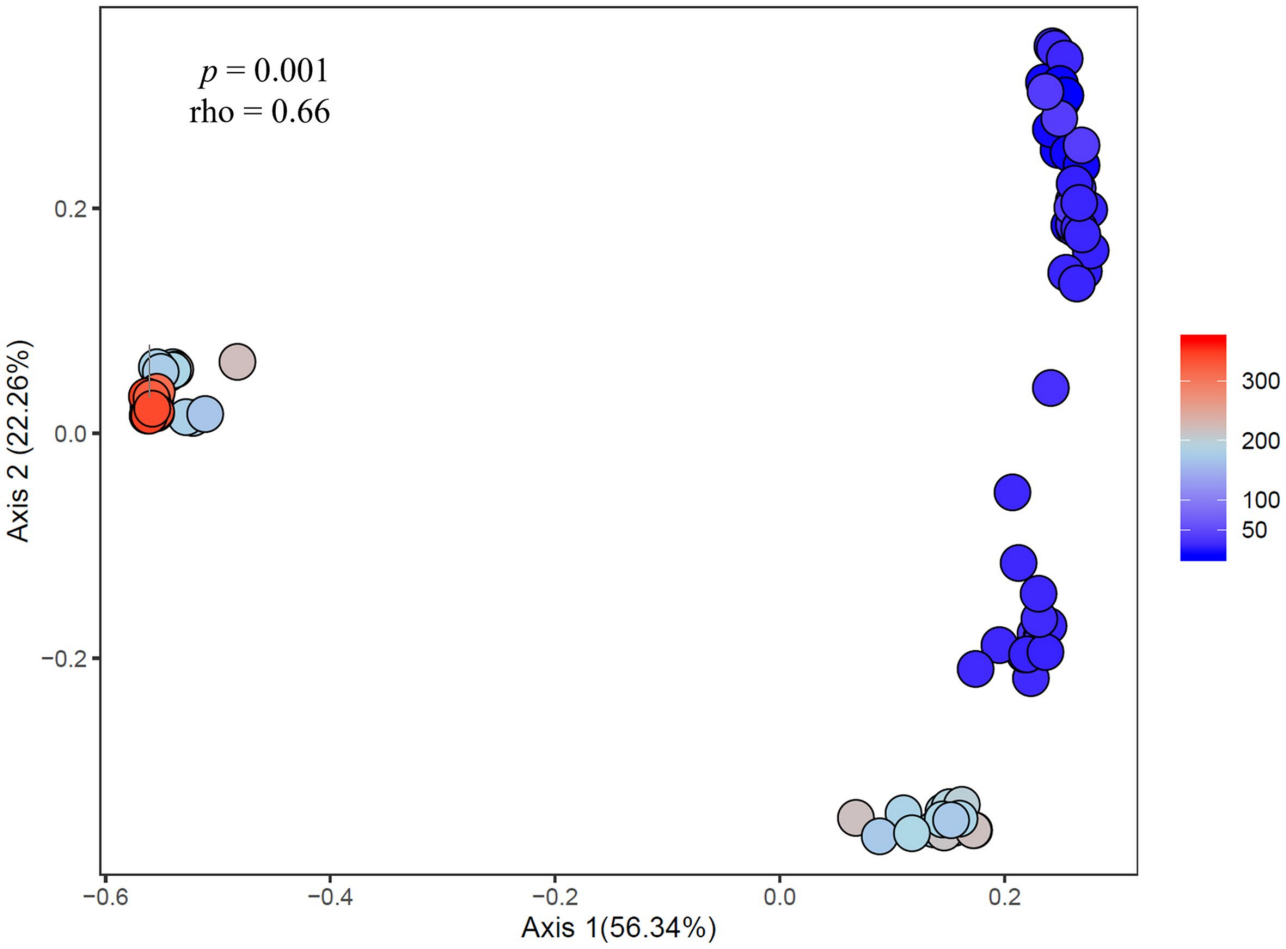
Principal coordinates analysis showing the dissimilarity of heterotrophic bacteria compositions (ASV-derived) between samples correlated to cyanobacteria abundance across experiments. Percent listed on the axes represents the percent variation explained. The color gradient denotes the fluoroprobe-derived cyanobacteria Chl *a* abundance per sample, with blue indicating lower cyanobacteria abundances and red indicating higher cyanobacteria abundances.

At the community level, the *Microcystis*-associated microbiomes were seemingly resilient to experimental perturbations. Across all experiments, treatment was not a significant explanatory variable of community variation, with no partitioning of samples by treatment in PCoA analysis of the beta diversities (*p* > 0.05; individual values of *p* listed in Supplementary Table 3; Supplementary Figure 3). Within individual experiments, however, experimental conditions significantly altered the bacterial communities (beta diversity) in each experiment (*p* = 0.001) but no individual treatment was statistically significant (Supplementary Table 2). There were similar clustering patterns of the treatments observed across experiments in accordance with the shifts observed in individual bacterial responses with nitrogen and temperature were most influential on the bacterial community structures (Figure 7). In both Lake Agawam experiments, the +T treatment communities were the most dissimilar among treatments (Supplementary Table 5), forming a separate cluster from the control, +N and +P samples (Figure 7) which accounted for around half of the community variation per experiment (51.2 ± 3.04%; PCoA axis 1). In the Lake Erie experiments, the +NT treatment communities were the most different from the control in all experiments except M1 (Supplementary Table 5), with the +T samples diverging from the control in the same direction largely along axis 1 in all four experiments (Figure 7). The +N samples also partitioned from the control cluster but in the opposite direction of the +T treatments along axis 1 in experiments M1 and M2 (Figure 7), with some of highest dissimilarities (%) across all experiments observed between the +N and +T/+NT communities (Supplementary Table 5). The divergent clustering of the +T/+NT and +N samples in all LE experiments (Figure 7) indicates that temperature and nitrogen were strong drivers of the community shifts with opposite effects and that temperature was the prime driver of the +NT interaction. The +P treatment was the least influential on the microbiome community structure (Supplementary Table 5) but did partition from the other treatments along PCoA axis 2 (17.9 ± 3.50%) in half of the experiments (Sept 26th, M1, M4; Figure 7). While treatment had an overall significant main effect on the community structure (Supplementary Table 2) there were no significant differences in diversity (alpha or beta) between individual treatments (*p* > 0.05; exact values listed in Supplementary Table 3) although the +N communities were generally the most even and rich (3 of 6 experiments) while the +T and +NT communities exhibited the greatest number of ASVs in 2 of 6 experiments (Supplementary Table 4). Variations in the microbiome community structure (beta diversities) were significantly correlated with cyanobacterial abundance, both between experiments and between treatments within experiments in four of six experiments (*p* < 0.05; individual values of *p* listed in Table 3, Figure 8, Supplementary Figure 4). Further, the degree of dispersion among the communities between treatments was related to cyanobacterial abundance, with higher abundance experiments being significantly less dispersed (*p* < 0.05; individual values of *p* listed in Supplementary Table 3), indicating cyanobacterial biomass had a greater organizing effect on bacterial communities than experimental treatments.

**Table 3:** Spearman’s rank-order correlations between fluorometrically quantified cyanobacterial biomass and the beta diversities of bacterial communities within the phycosphere of *Microcystis* colonies for each experiment and all experiments.

Experiment	Sample size	Permutations	Alternative hypothesis	Spearman rho	Value of p
All	84	999	two-sided	0.6627	**0.001**
July 24th	12	999	two-sided	0.3069	**0.05**
Sept 26th	12	999	two-sided	0.0057	0.962
LEM1	15	999	two-sided	0.3349	**0.014**
LEM2	15	999	two-sided	0.2244	**0.041**
LEM3	15	999	two-sided	−0.0541	0.765
LEM4	15	999	two-sided	0.2834	**0.006**

Bolded values denote statistically significant correlations.

### Predicted Metagenomes of the *Microcystis* Microbiome

In parallel with the 16S-rRNA derived community compositions, the taxonomically inferred predicted metagenomes of the *Microcystis* microbiomes significantly differed between all experiments (*p* = 0.001, Supplementary Table 6). While the taxonomic compositions clustered primarily by lake source, the variation among the predicted metagenomes was largely explained by the associated cyanobacterial abundance, with experiments separated by increasing cyanobacterial levels from right to left along PCoA axis 1 (70.31%; Supplementary Figure 5). Lake source was the second most explanatory variable, accounting for around 19% of the variation along axis 2 (Supplementary Figure 5), with each lake having significantly different predicted metagenomes (*p* = 0.001, Supplementary Table 6). There was a significant effect of treatment on the predicted metagenomes in five of six experiments (*p* < 0.01, all but July 24th, individual values of *p* listed in Supplementary Table 6) with shared patterns of divergence among the treatments across experiments (Supplementary Figure 6). These patterns mimicked those of the taxonomic compositions but with generally weaker dissimilarities (Supplementary Table 5) with less distinct separation of treatments as no individual treatment significantly altered potential metagenomes within any individual experiment (Supplementary Figure 6). Across experiments, the +T and +NT predicted metagenomes were among the most dissimilar from the other treatments in the Lake Agawam and Lake Erie experiments, respectively (Supplementary Figure 6; Supplementary Table 5). Further, in three of four Lake Erie experiments the +T and +N samples diverged from the control in a similar direction as the +NT treatment (all but M4) while the +N samples formed a separate cluster, largely in the opposite direction of the +T treatments (all but M3; Supplementary Figure 6). The +P treatment was the least influential on the predicted metagenomes (Supplementary Figure 6; Supplementary Table 5).

Among bacterial genes involved in nutrient acquisition and metabolism, several were present in high abundances within experiments, particularly in experiments exhibiting higher cyanobacteria levels (LA experiments and LE M2, M3). Genes involved in ammonium acquisition (*cynT*, *gcvT*, *amt*) glutamate synthesis (*gltB*, *glnA*, *glnB*), and alkaline phosphatase activity (*pho* gene family) were highly abundant in these experiments. Within experiments, treatments altered the predicted relative abundance of genes (KOs) involved in N and P cycling (Supplementary Figure 7), with 15–40% of the total predicted genes found to be significantly differentially abundant between treatments (*p* < 0.05, effect size >0.8). In the LA experiments, of the 17 and 40% significantly altered (*p* < 0.05) genes in the July 24th and Sept 26th experiments, those involved in N and P cycling were most influenced by the +T treatment compared to the control (Supplementary Figure 7) as genes involved in nitrate reduction, denitrification, and nitric oxide reductase (*narGHIJVWYZ*, *nxrAB*, July 24th only: *nirBD*, Sept 26th only: *nirK*, *norB* and *nosDZ*) became significantly less abundant in +T (*p* < 0.05 for all; Supplementary Figure 7). Genes involved in ammonium assimilation had a more varied response to the treatments, with significantly reduced abundances in the +N and +T treatments in the July 24th experiment (*amt*, *glnG*, *ntrC*), but several increased (*gcvT*, *glnABE*) and several decreased (*glnKL*, *ntrB*, *gltB*) due to +T in the Sept 26th experiment (*p* < 0.05 for all; Supplementary Figure 7). The +P treatment significantly (*p* < 0.05) decreased the predicted abundance of denitrification genes (*nirK*, *norB*, *nosDZ*) and increase in ammonium assimilation genes (*glnEKL*, *nirB*) in the Sept 26th experiment (Supplementary Figure 7). Three phosphatase genes involved in P cycling were also significantly (*p* < 0.05 for all) altered in the Sept 26th experiment, primarily being enriched in the +T treatment (*phoRPB1*; Supplementary Figure 7).

In the Lake Erie experiments, the +N and +NT treatments were highly and differentially influential on N cycling genes (Supplementary Figure 7). The M1 experiment predicted metagenomes were most impacted by treatments, with ammonium assimilation (*gcvT*, *glnAG*, *ntrC*, *gltBD*) and urease (*ureABCDFGH*) genes being significantly enriched and dissimilarity nitrate reduction, denitrification and ammonification genes (*napABCD*, *narGHIJPQVWYZ*, *nrxAB*, *nirBD*, *nrfACDFG*, *norV*) being the mostly significantly reduced in the +N compared to the control (*p* < 0.05 for all; Supplementary Figure 7). In contrast, ammonium assimilation (*amt*, *gvcT*, *gltBD*) and denitrification (*nirBDK*) genes were significantly depleted in +NT treatment (*p* < 0.05 for all; Supplementary Figure 7). Only two P cycling genes (*phoB/phoD*) significantly differed in abundance (*p* < 0.05 for all), being enriched in +N and depleted in +T and +NT treatments. In the M2 experiment, N cycling genes in the denitrification (*nir*) and nitric oxide synthase (*nos*) families were significantly (*p* < 0.05 for all) depleted in the +N treatments but significantly (*p* < 0.05 for all) enriched in +T (*nir* only), while nitrate reductase (*nar*) and urease (*ure*) genes were significantly (*p* < 0.05 for all) enriched in +N treatment but depleted in the +T and +NT treatments (Supplementary Figure 7). Individual phosphatase genes had varied response to the treatments, with *phoP*, *phoB1* and *phoR* genes significantly (*p* < 0.05) depleted in N and enriched in +T and +NT (Supplementary Figure 7). Responses were muted in the M3 and M4 experiments (Supplementary Figure 7).

Finally, there were also significant differences detected in the cumulative relative abundances of bacterial taxa with known *nifH* (nitrogen fixation) and *phoX* (alkaline phosphatase) genes in the NCBI database across treatments. Specifically, there was a significant main effect of treatment on the cumulative relative abundance of the *nifH* and *phoX*-containing bacteria in the M1 (*p* = 0.0001, 4.7E-5), M2 (*p* = 7.16E-5, 9.1E-6) and M3 (*p* = 0.0009, 0.0019) Lake Erie experiments. Across individual treatments, the relative abundance of *nifH*-containing bacteria genera was significantly enriched in the +N treatments compared to the control in M1 (*p* < 0.001) and M2 (*p* = 0.005) but significantly depleted in the +NT treatment compared to the control in experiment M3 (*p* = 0.011; Supplementary Figure 8). The cumulative relative abundance of *phoX*-containing bacteria was also significantly enriched in the +N treatments the M1 (*p* = 0.001) and M2 experiments (*p* = 0.003) and significantly depleted by the +T (*p* = 0.005) and +NT (*p* = 0.008, 0.03) treatments in the M2 and M3 experiments (Supplementary Figures 8, 9).

## Discussion

Recently, ecosystem studies of the microbiota associated with *Microcystis* blooms and colonies have identified the conserved community structure shared across geographically and temporally distinct blooms (Cook et al., 2020; Jankowiak and Gobler, 2020; Smith et al., 2021). The conserved molecular functionality of these microbes may increase *Microcystis*’ robustness to environmental variation, thus promoting its prolific nature (Jankowiak and Gobler, 2020). Still, the effects of environmental perturbations on the endosymbiotic microbial communities within *Microcystis* colonies is unclear. Here, we examined the effects of nutrient availability and temperature on the microbiomes from *Microcystis* colonies to provide insight into the dynamic nature of these endosymbiont and how they may influence blooms.

### The *Microcystis* Microbiome Is Resistant to Environmental Drivers

Our results strongly indicate that *Microcystis*-associated microbial communities were fairly resistant to the environmental perturbations. First, the majority of ASV taxa identified during this study were shared across treatments belonging to the core microbiome, with only rare taxa unique to individual treatments suggesting limited exchange (loss/colonization) of taxonomic groups due to the treatments. Further, at the community scale there were not significant differences among the treatments in any of the experiments in terms of both diversity and predicted functional potential. Additionally, variations in the microbiome community structure (beta diversities) and the degree of dispersion among the microbial communities between treatments were significantly altered by cyanobacterial abundance, rather than individual treatments, suggesting the treatments were less influential than the intensity of the blooms. These findings are consistent with the conserved compositional patterns observed among field populations spanning vast differences in hydrodynamic, physical and chemical conditions and suggest a greater degree of host-mediated selection than external environmental selection on the microbiome community (Liu et al., 2019; Cook et al., 2020; Jankowiak and Gobler, 2020; Smith et al., 2021), promoting the net growth of select bacterial groups. *Microcystis* and other cyanobacteria have been shown to have the ability to alter prokaryotic communities through the release of bioactive and nutritional compounds (Casamatta and Wickstrom, 2000; Wang et al., 2017; Chia et al., 2018; Hoke et al., 2021). For example, *Microcystis* exudates have been shown to attract and enhance the growth of select bacteria, with greater chemotaxis toward exudates by bacteria found in association with *Microcystis* colonies compared to non-associated bacteria (Casamatta and Wickstrom, 2000). Further, genome sequencing of bacteria isolated from the *Microcystis* phycosphere identified genes used in signaling and nutrient exchange between bacteria and *Microcystis* (Hoke et al., 2021). The greater host influence than environmental influence on the microbiome composition seen here is consistent with an eight-month study of *Microcystis* blooms that found microbiomes exhibited significantly less seasonal variation than the free-living communities, presumably due to the presence of host selective pressures (Jankowiak and Gobler, 2020).

During experiments, there was greater similarity among the predicted metagenomes than taxonomic composition across treatments suggesting that metabolic functionality was more resistant to environmental perturbation than microbial diversity. While the metagenomic analyses used in this study were predictive, this pattern was consistent with an emerging body of evidence describing relatively consistent metabolic potentiality of collective *Microcystis* microbiomes, despite the potential dynamic phylogenetic composition (Steffen et al., 2014; Li et al., 2018; Smith et al., 2021). These findings suggest there is environmental selection for bacteria with distinct metabolic characteristics and further suggests that function provided by symbionts may be more influenced by selection than their taxonomy. This may account for the similar responses in compositional shifts to the treatments seen across experiments, such as the consistently divergent responses to N and elevated temperature treatments, despite significantly different taxonomic compositions between experiments. The greater taxonomic shifts between treatments within experiments compared to potential biochemical functional changes may also result from differential growth among bacteria that exhibit similar metabolic capabilities but are differentially adapted to environmental conditions. Together these findings emphasize the need to consider function in addition to taxonomic composition when considering the role of the microbiome in impacting *Microcystis* blooms.

While the experimental elevation of N, P and temperature did not cause significant alterations in the collective composition of the *Microcystis*-associated microbiomes at the community scale, significant changes in abundance of numerous individual taxa were observed. The most influential treatments on bacterial taxa were the N and elevated temperature treatments, which also caused the greatest increase in cyanobacteria and *Microcystis* biomass across experiments, partly accounting for the significant positive correlation between beta diversity and cyanobacterial biomass. It has been postulated that the microbiome acts as a functional community from which bacteria can be promoted under select conditions when their metabolic capabilities are favored, for example, high affinity nutrient acquisition strategies being favored under nutrient deplete conditions (Costello et al., 2012; Zeng et al., 2017). Our findings of differential taxonomic shifts in response to the treatments despite conserved collective microbiome composition and the association with enhanced *Microcystis* growth support this theory. While shifts in abundance of individual taxa within the microbiome may have contributed to increased cyanobacteria abundance, elevated N and temperature can promote *Microcystis* growth in axenic cultures (Reynolds, 1984; Paerl et al., 2011; Harke et al., 2015), suggesting changes in cyanobacterial abundance may have been caused directly by the environmental factors, indirectly by changes in bacterial taxa, or, more likely, by both processes. Similarly, changes in microbial abundances may have been caused by shifts in N and temperature, by increased cyanobacterial growth causing different environmental conditions within *Microcystis* colonies (e.g., increased release of organic matter, other nutritional factors, or allelopathic compounds), or both processes. Consistent with this concept, Hoke et al. (2021) sequenced the genomes of bacteria isolated from *Microcystis* colonies and found that genes encoding for the utilization of algal organic carbon in their genomes and expressed during blooms in Lake Erie. Regardless, it is clear that elevated levels of N and temperature increased cyanobacterial biomass as well as the relative abundance of select bacterial taxa within *Microcystis* colonies.

Among the environmental factors explored in this study, temperature had the largest effect on microbial community diversity and potential functionality within *Microcystis* colonies, while the effects of N were less impactful and the opposite of elevated temperatures. This indicates that N and temperature had differential impacts on microbial community structure. Temperature is a well-known regulator of enzymatic activity and microbial communities (Jones, 1977; Pomeroy and Wiebe, 2001; Sunagawa et al., 2015) and increasing temperatures are known to reduce microbial diversity in aquatic ecosystems (Sunagawa et al., 2015). While P has been long thought to be the main factor that controls the growth of cyanobacteria and heterotrophic bacteria in freshwater ecosystems (Schindler, 1977; Smith and Prairie, 2004; Schindler et al., 2016), the critical role of N in driving non-diazotrophic CHABs, such as those caused by *Microcystis*, has been established (Conley et al., 2009; Gobler et al., 2016; Paerl et al., 2016). N may be an even more important driver of microbial communities within *Microcystis* colonies, where the enrichment of carbon-rich extracellular polysaccharides creates an increased stoichiometric demand for N (Van de Waal et al., 2010) and the three-dimension structure and diurnal pulses in photosynthetic oxygen may facilitate coupled nitrification-denitrification (An and Joye, 2001; Jankowiak and Gobler, 2020) and thus nocturnal loss of N from colonies.

When exposed to both increased N and temperature, endosymbiotic communities within colonies responded similarly to the elevated temperature treatment and in many cases had a more extreme shift, affirming the importance of temperature as a driver of microbial communities. This was seen at the community level, but also at the taxon level. For example, elevated temperatures caused a significant reduction in the relative abundance of Alphaproteobacteria and Betaproteobacteria in two of four Lake Erie experiments, while the combination of elevated N and elevated temperatures did so in three of the Lake Erie experiments while also significantly lowering the relative abundances of Bacteroidetes in the same three experiments. While some taxa significantly increased in abundance due to increased N availability (e.g., Armatimonadetes, Alphaproteobacteria, and *Bacillus*), the trends were less consistent across experiments. This suggests that while N is an important driver of these communities, temperature was a stronger driver perhaps since it a central regulator of cellular metabolism. These findings also suggest that as peak seasonal temperatures continue to increase this century (IPCC, 2021) and nutrient loading rates are altered (Ho et al., 2017), microbial communities associated with *Microcystis* colonies are also likely to display differential responses to these contrasting drivers. Importantly, this study examined trends in the relative, and not absolute abundances, of taxa as bacterial abundances were not quantified. Thus, it is possible that increases or decreases in relative abundance of a given taxa may have been caused in changes in the absolute abundance of that taxa, or by the changes in the growth and abundance of other taxa.

Environmental perturbations had significant impacts on some predicted gene sets associated with N and P cycling. For example, the abundance of *nifH*, encoding for nitrogenase, *ure*, encoding urease, and *phoX*, encoding for alkaline phosphatase, significantly increased under elevated N conditions in multiple Lake Erie experiments. In contrast, *nifH* and *phoX* significantly decreased under elevated temperatures, even when combined with elevated N in Lake Erie. Further, elevated N reduced the abundance of denitrification genes in half of the Lake Erie experiments, while elevated temperature decreased the abundance of phosphates and nitrate reductases in two experiments. Collectively, these findings demonstrate that some functional capabilities of microbial communities associated with N and P cycling within *Microcystis* colonies were shifting in response to prevailing environmental conditions. Pulses of N have been shown to promote cyanobacterial and bacterial growth, biomass (Gobler et al., 2016; this study), and thus community N and P demand, necessitating increased nitrogen fixation (*nifH*), urease activity (*ure*), and hydrolysis of phosphomonoesters (*phoX*; Dyhrman et al., 2007). Higher temperatures are likely to increase the rates of growth, metabolic activities, and nutrient assimilation of some cyanobacteria and bacteria (Jones, 1977; Pomeroy and Wiebe, 2001; Paerl et al., 2011), perhaps driving microbial communities into an even greater nutrient deficit and state of physiological impairment, therefore, potentially reducing the abundance of microbes with the ability to carry out nitrogen fixation and P scavenging, accounting for the lowered *nifH* and *phoX* abundances under elevated temperatures. Alternatively, the microbes that perform these biochemical functions may simply be poorly adapted to sudden rises in temperature. Regardless, the dynamics nature of *nifH* abundances within *Microcystis* colonies in response to environmental drivers suggests diazotrophy may represent be an important supply of N to these populations that may support the proliferation of blooms, particularly during periods of N scarcity.

Of the environmental factors considered in this study, P was the least influential environmental variable, having the smallest effect on cyanobacteria and bacterial abundance and community composition, being the most similar to the control across all experiments, and not altering the abundance of any of the N and P cycling genes. In some respects, this might be considered surprising given that P has been previously shown to limit (DeBruyn et al., 2004; Stumpf et al., 2012; Michalak et al., 2013) or co-limit (North et al., 2007) the abundance of plankton communities in Lake Erie. A series of observations since those studies, however, have identified the key role N can play in controlling cyanobacterial blooms in western Lake Erie during late summer (Chaffin et al., 2013; Chaffin and Bridgeman, 2014; Davis et al., 2015; Gobler et al., 2016; Jankowiak et al., 2019; Newell et al., 2019; this study). Regardless, like *Microcystis* (Harke et al., 2012), a high percentage of the microbial population was predicted to possess a suite of phosphatases and high affinity P transporters, making them well-adapted to low P conditions (Dyhrman et al., 2007). Still, in Lake Erie, there were specific taxa that were responsive to elevated P conditions. For example, the relative abundance of Firmicutes significantly increased in two of four experiments within the elevated P treatments, while the relative abundance of Alphaproteobacteria and Betaproteobacteria decreased within the elevated P treatments in two of four experiments. Hence, while P did not cause a restructuring of *Microcystis* microbiomes, there were taxa that were responsive to this nutrient.

## Conclusion

During this study, elevated N, and to a lesser extent elevated temperature, were found to consistently and significantly promote the intensity of *Microcystis*-dominated cyanobacterial blooms in two North American lakes. In contrast, the diversity of microbiome of *Microcystis* colonies was more influenced by the intensity of cyanobacterial blooms and biomass than by the same experimental environmental perturbations. Minor, but significant, changes were observed among some endosymbiotic microbial taxa in responses to temperature, and to a lesser extent, N, while responses of predicted metagenomes were more muted than those of microbial taxa, suggesting a resilience of physiological function across *Microcystis* microbiomes. Among predicted gene sets associated with microbes, some associated with N and P cycling were significantly altered, with the *nifH*, *ure*, and the *phoX* genes displaying the most consistent and significant trends. Collectively, these findings are consistent with the temporal and spatial consistency previously noted among *Microcystis* microbiome (Jankowiak and Gobler, 2020) and suggests that the environment within the *Microcystis* phycosphere is resilient to environmental perturbation and that bacteria providing essential functions are maintained. This consistency of microbial communities and function may support the ability of *Microcystis* to persist through both favorable and unfavorable short-term fluctuations in environmental conditions. While the duration (48 h) and the levels of nutrients and temperature used in our experiments have been previously shown to significantly alter bacterial densities (Del Giorgio et al., 1997; Gobler et al., 2008; Taylor and Cunliffe, 2017), it is likely that more sustained and/or intense exposure to environmental disturbance would have elicited a stronger organizing effect on endosymbiotic microbial communities within *Microcystis* colonies. Due to the high complexity of these interactions, additional studies of the *Microcystis* phycosphere across a greater range and diversity of geographic and environmental settings are needed, as are studies examining how these drivers effect colonial rate processes and biochemical activities.

## Author’s Note

The ecological relationship between *Microcystis* and its associated microbiome may influence cyanobacteria bloom ecology. While previous studies have characterized microbial communities associated with *Microcystis*, the current study demonstrates that the drivers of *Microcystis* blooms differ from those of endosymbiotic bacteria within *Microcystis* colonies. While *Microcystis* populations were primarily controlled by nitrogen, temperatures were a stronger driver of endosymbiotic microbial communities. While the relative abundance of some individual bacteria taxa was altered by experimental conditions, community diversity was not but rather was inversely correlated with the intensity of *Microcystis* blooms. While predicted metabolic function of bacteria within the phycosphere was minimally impacted by environmental drivers, the predicted abundance of nitrogenase (*nifH*), alkaline phosphatase (*phoX*), and urease (*ure*) genes significantly increased in response to N but decreased in response to increased temperature. Collectively, the resilience of microbial community structure and function within colonies suggests they may support the ability of *Microcystis* to persist through short-term fluctuations in environmental conditions.

## Data Availability Statement

The data sets presented in this study can be found in online repositories. The names of the repository/repositories and accession number(s) can be found in the article/Supplementary Material.

## Author Contributions

JJ and CG conducted the research and contributed to the writing of this article. All authors contributed to the article and approved the submitted version.

## Funding

This work was supported by the NOAA-MERHAB program (publication number 240) and the Simons Foundation.

## Conflict of Interest

The authors declare that the research was conducted in the absence of any commercial or financial relationships that could be construed as a potential conflict of interest.

## Publisher’s Note

All claims expressed in this article are solely those of the authors and do not necessarily represent those of their affiliated organizations, or those of the publisher, the editors and the reviewers. Any product that may be evaluated in this article, or claim that may be made by its manufacturer, is not guaranteed or endorsed by the publisher.

## Footnotes

^1^ https://software.broadinstitute.org/morpheus
