# Supplementary material for: Dynamic Responses of Endosymbiotic Microbial Communities Within Microcystis Colonies in North American Lakes to Altered Nitrogen, Phosphorus, and Temperature Levels
Source: Front Microbiol. 2022 Feb 10;12:781500. doi: 10.3389/fmicb.2021.781500 (PMC8867038; doi:10.3389/fmicb.2021.781500)
Supplement: Supplementary file 1 [file Data_Sheet_1.PDF]

Supplemental Figures and Tables for

Dynamic responses of endosymbiotic microbial communities within *Microcystis* colonies in North American lakes to altered nitrogen, phosphorus, and temperature levels

Christopher J. Gobler[1*], Jennifer G. Jankowiak[1]

[1]Stony Brook University, School of Marine and Atmospheric Sciences, Southampton, NY, USA

*Christopher.gobler@stonybrook.edu

SUPPLEMENTARY FIGURES

Supplementary Figure 1: 16S rRNA derived relative abundances of cyanobacteria genera in the A) LA July 24th, B) LA September 26th, C) LE M1, D) LE M2, E) LE M3, and F)LE M4 experiments. Arrows indicate significant enrichment (up) or depletion (down) of the class in each treatment (CTR= Control, +N = Nitrate addition, +P = Orthophosphate addition, +T = elevated incubation temperature) compared to the control.

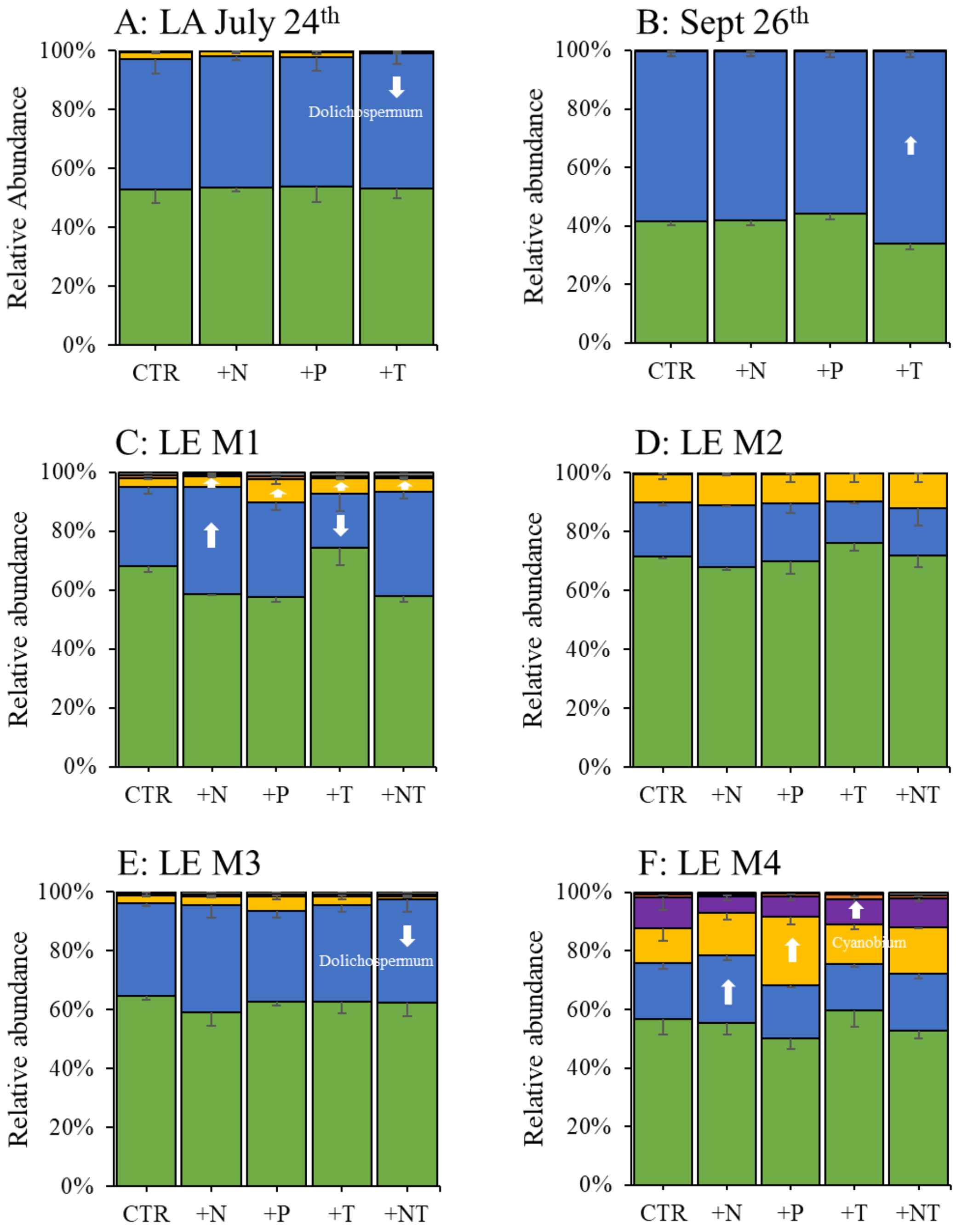

Supplementary Figure 2: Proteobacterial class relative abundance in the A) LA July 24th experiment, B) LA September 26th experiment, C) LE M1 experiment, D) LE M2 experiment E) LE M3 experiment and F) LE M4 experiment. Arrows indicate significant enrichment (up) or depletion (down) of the class in each treatment (CTR= Control, +N = Nitrate addition, +P = Orthophosphate addition, +T = elevated incubation temperature) compared to the control.

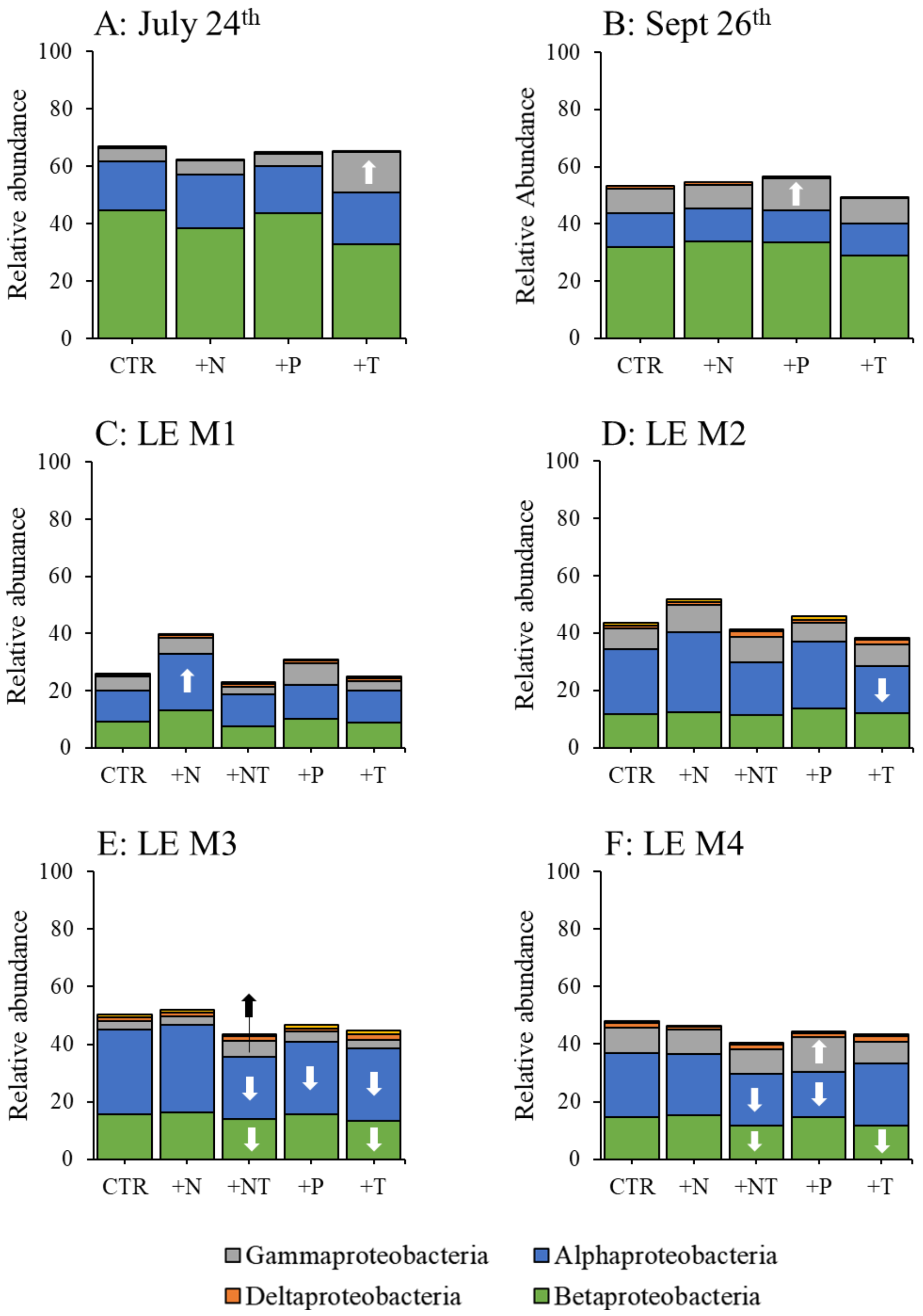

Supplementary Figure 3: Principal coordinates analysis (PCoA) conducted on ASV abundances showing the dissimilarity of heterotrophic bacteria compositions between samples from all Lake Agawam and Lake Erie experiments. Color denotes the A) experiment and B) treatment (CTR= Control, +N = Nitrate addition, +P = Orthophosphate addition, +T = elevated incubation temperature). Percent listed on the axes represents the percent of variation explained by PC1 and PC2.

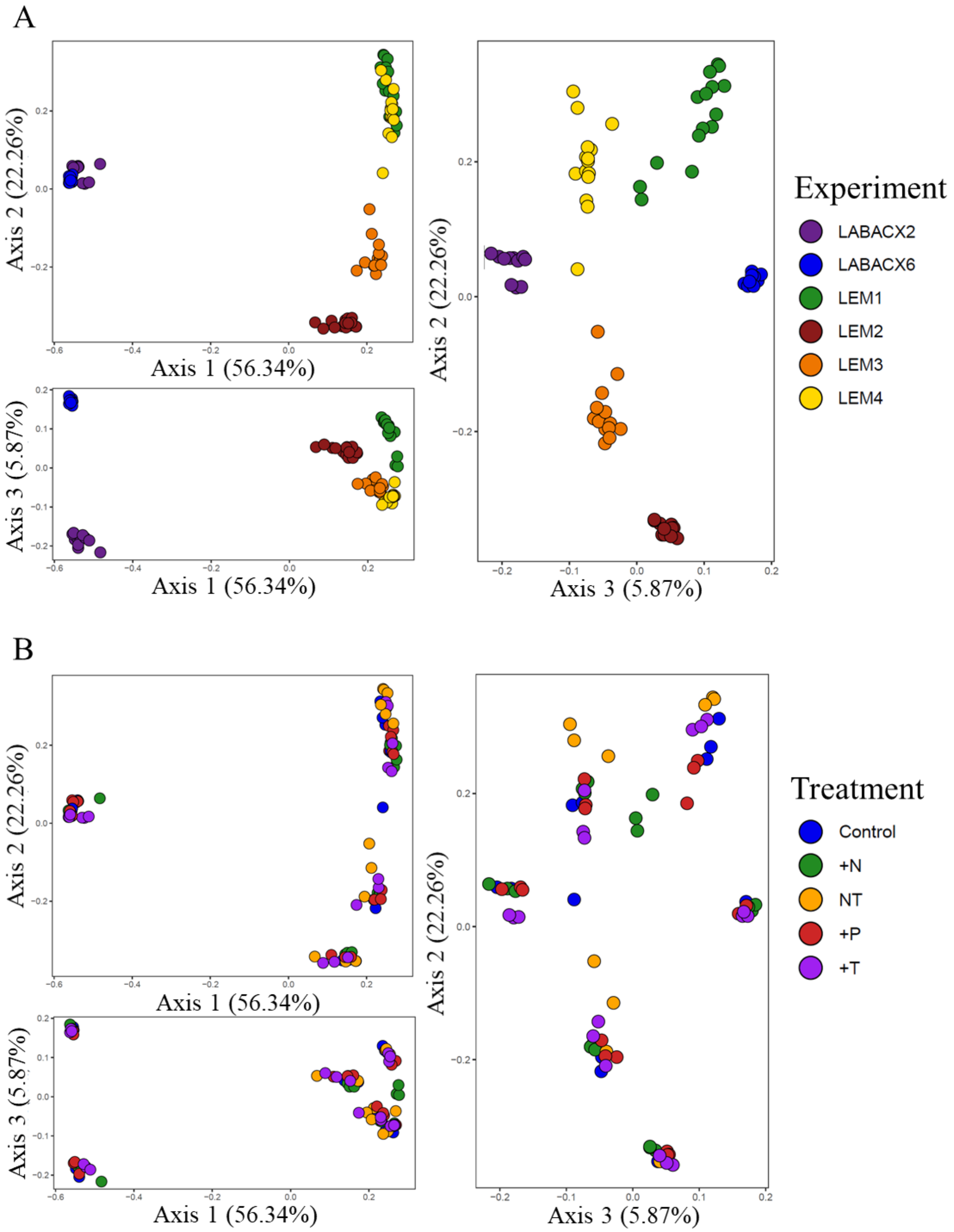

Supplementary Figure 4: Principal coordinates analysis (PCoA) showing the dissimilarity of heterotrophic bacteria compositions (ASV-derived) between samples correlated to cyanobacteria abundance in the in the A) LA July 24th, B) LA September 26th, C) LE M, D) LE M2, E) LE M3 and F) LE M4experiments. Percent listed on the axes represents the percent variation explained. The color gradient denotes the fluoroprobe-derived cyanobacteria Chl *a* abundance per sample, with blue indicating lower cyanobacteria abundances and red indicating higher cyanobacteria abundances.

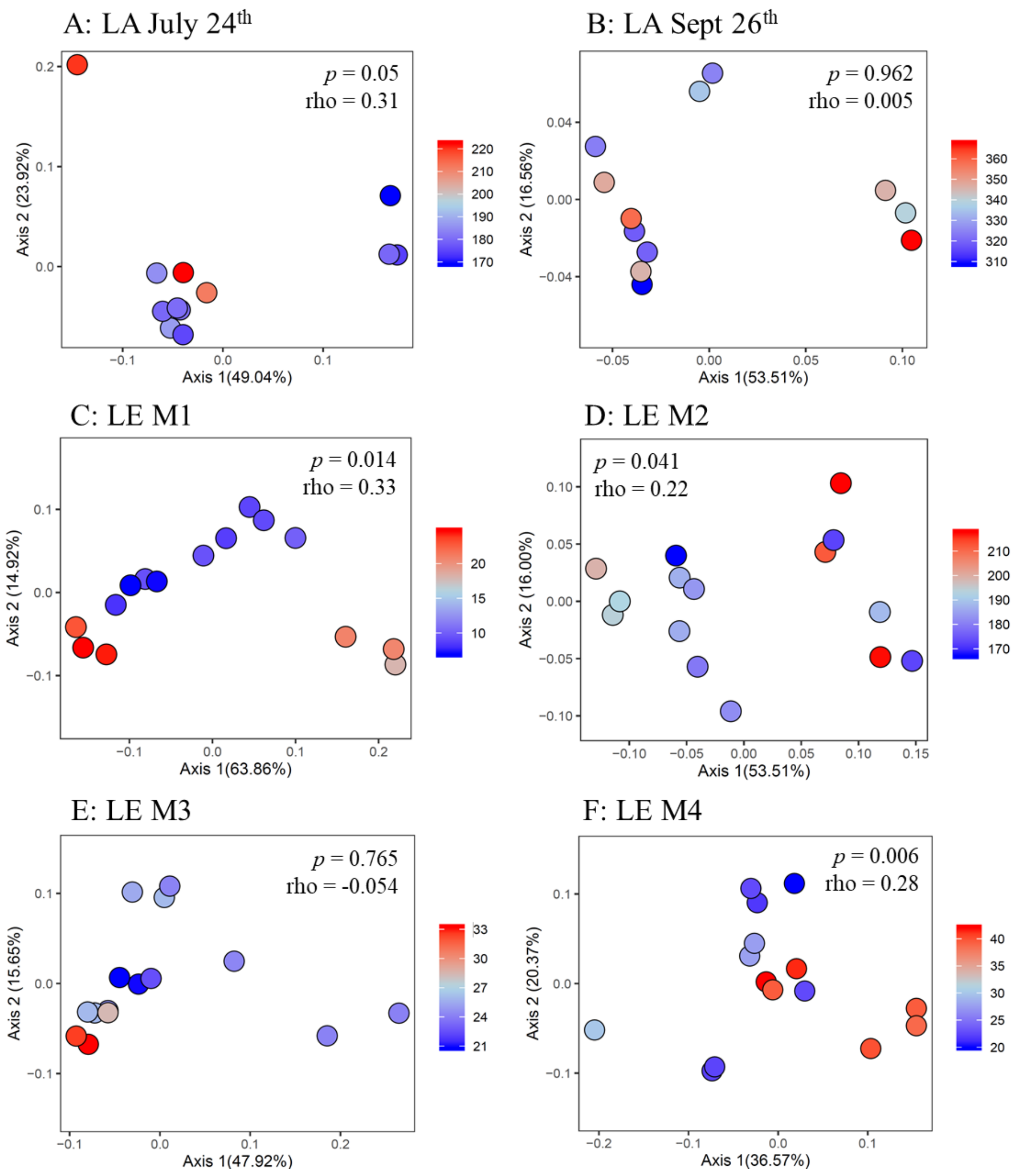

Supplementary Figure 5: Principal coordinates analysis (PCoA) conducted on PICRUSt predicted metagenomes showing the dissimilarity of the KEGG gene (KOs) compositions between samples in all Lake Agawam and Lake Erie experiments. Color denotes the experiment. Percent listed on the axes represents the percent of variation explained by PC1 and PC2. A-F) Dendrograms showing similarity of PICRUSt predicted metagenomes between samples in each experiment

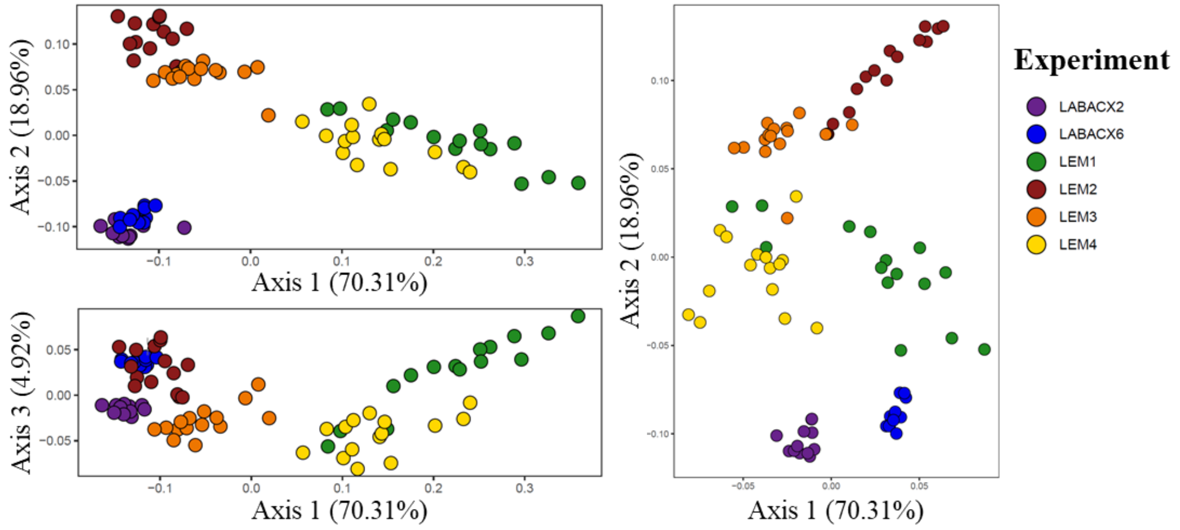

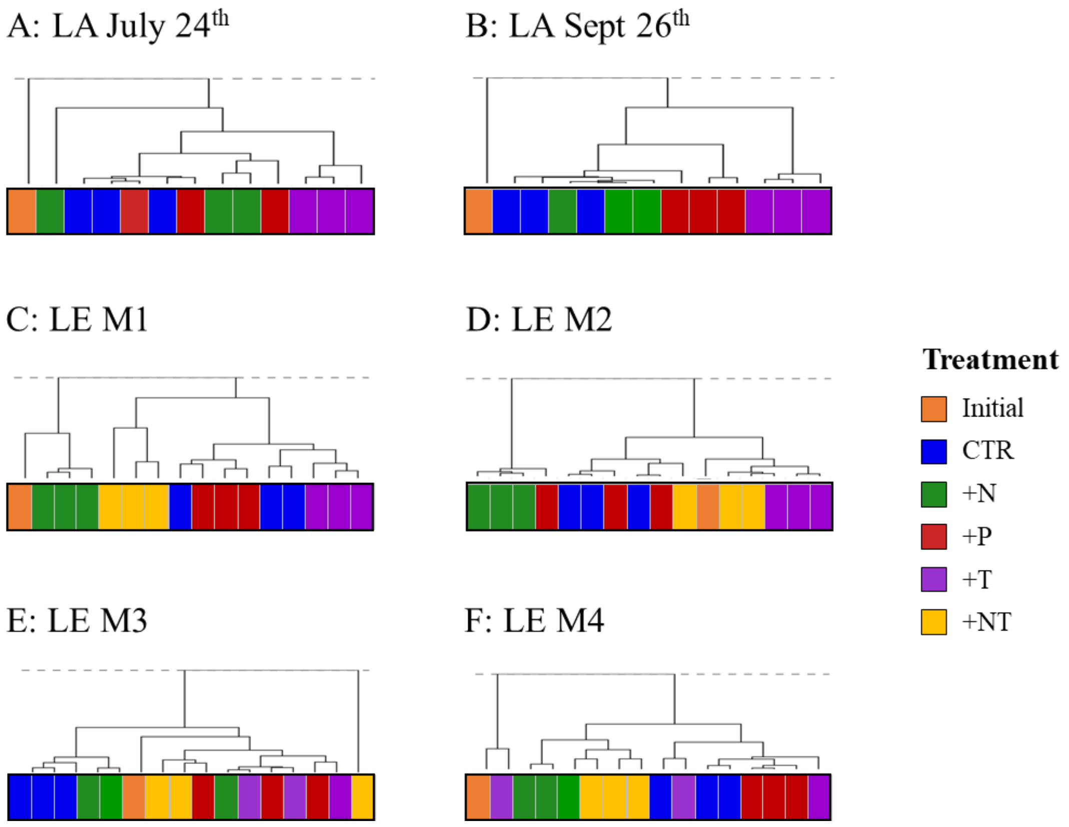

Supplementary Figure 6: Principal coordinates analysis (PCoA) conducted on PICRUSt predicted metagenomes showing the dissimilarity of the KEGG gene (KOs) compositions between samples in the A) LA July 24th, B) LA September 26th, C) LE M, D) LE M2, E) LE M3 and F) LE M4experiments. Color denotes the sample treatment (CTR= Control, +N = Nitrate addition, +P = Orthophosphate addition, +T = elevated incubation temperature). Percent listed on the axes represents the percent of variation explained by PC1 and PC2. P value indicates the significance of the main effect of treatment on the communities determined via PERMANOVA.

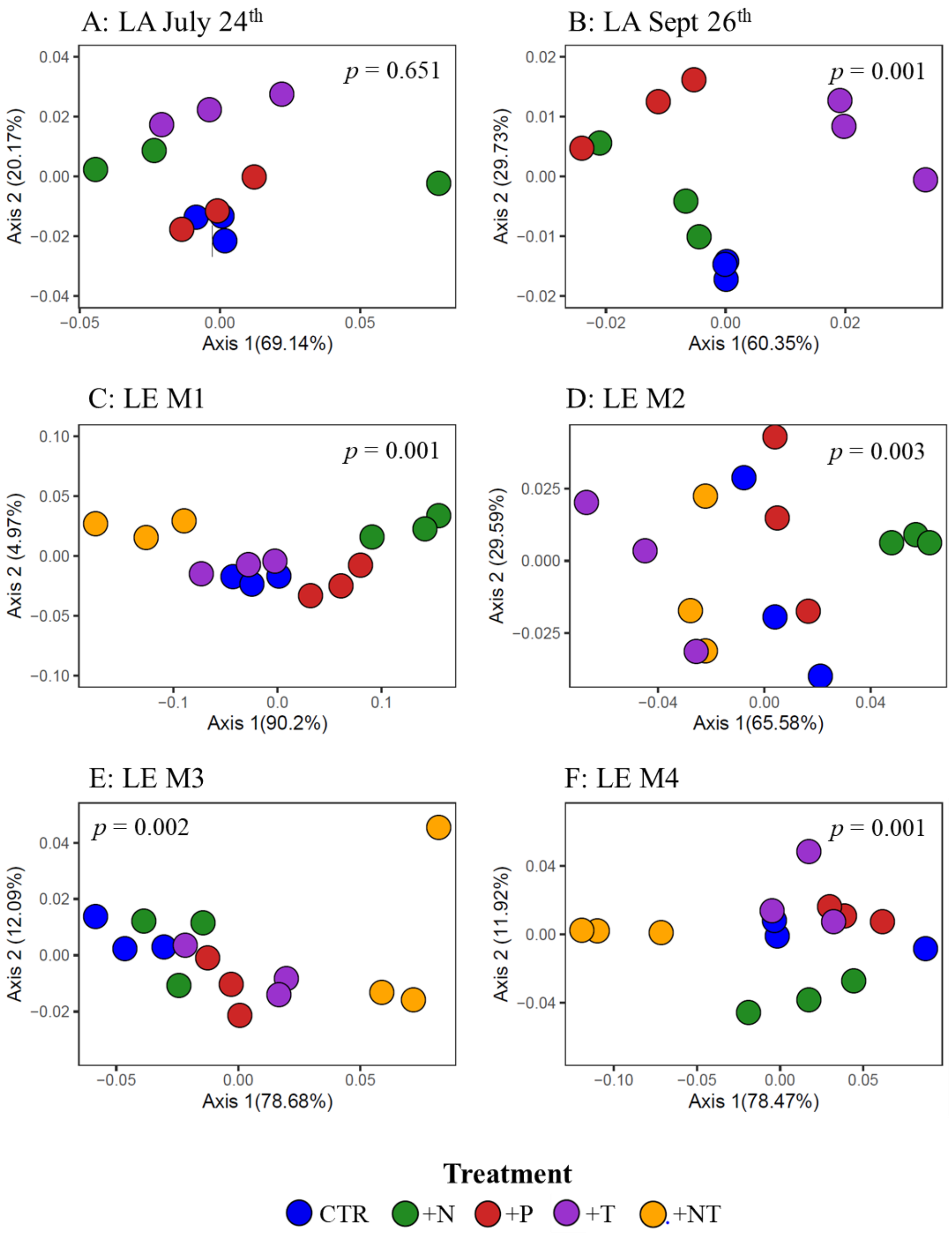

Supplementary Figure 7: Heatmap of PICRUST predicted abundances of genes (KOs) belonging to N and P cycling pathways found to be significantly ($p<0.05$) differentially abundant between treatments within experiments, analyzed via STAMP software (ANOVA with Tukey post hoc analysis and Benjamini-Hochberg FDR correction). Stars indicate treatments that significantly differed from the control and only genes with an effect size >0/8 are shown. Color denotes the mean relative abundance (row-wise) of genes per treatment, and KOs with the same abundance patterns and functions have been dereplicated into a single row and indicted in the gene column. The pathway column indicates the pathways KOs are involved in as per KEGG database , abbreviated as follows: AA: Ammonia assimilation, AM: ammonification, DNR: Dissimilatory Nitrate Reduction, DEN: Denitrification, NRR: N response regulator, BIO: N containing amino acid synthesis, CH: Cyanate hydrolysis, NS: Nitrosative stress, URE: urease, CA: Carbonic anhydrase, ANR: Assimilatory nitrate reduction, PHO: phosphatase.

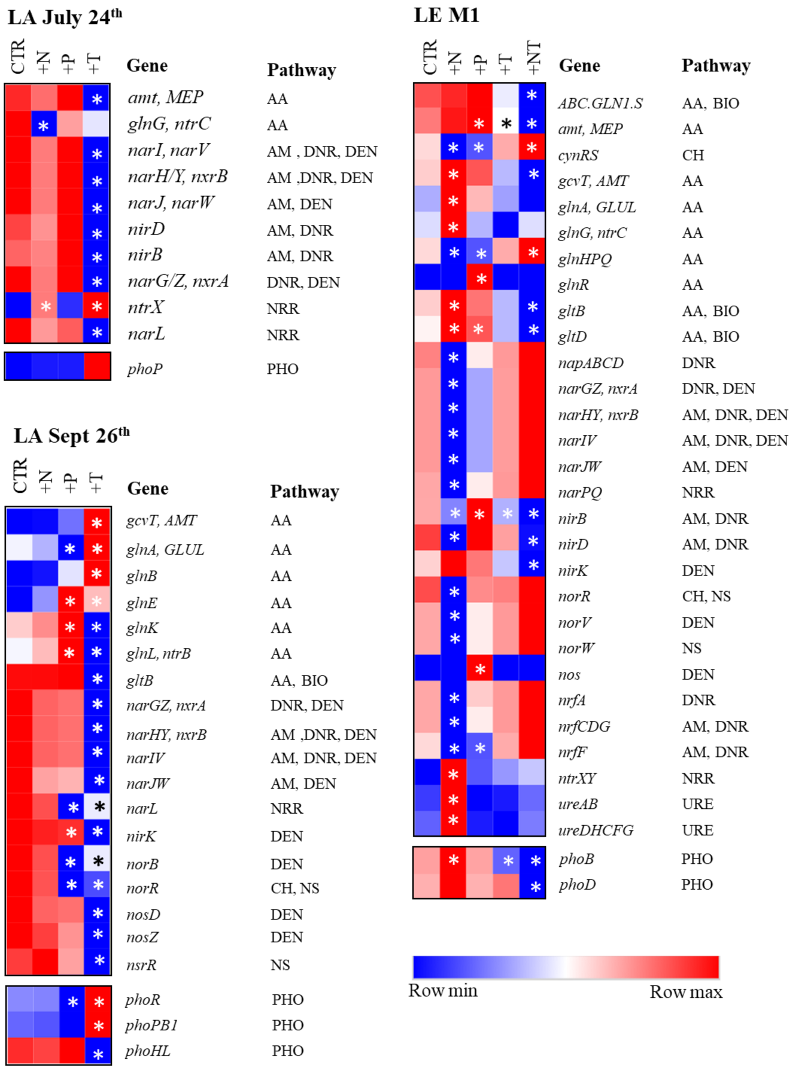

Supplementary Figure 7 continued:

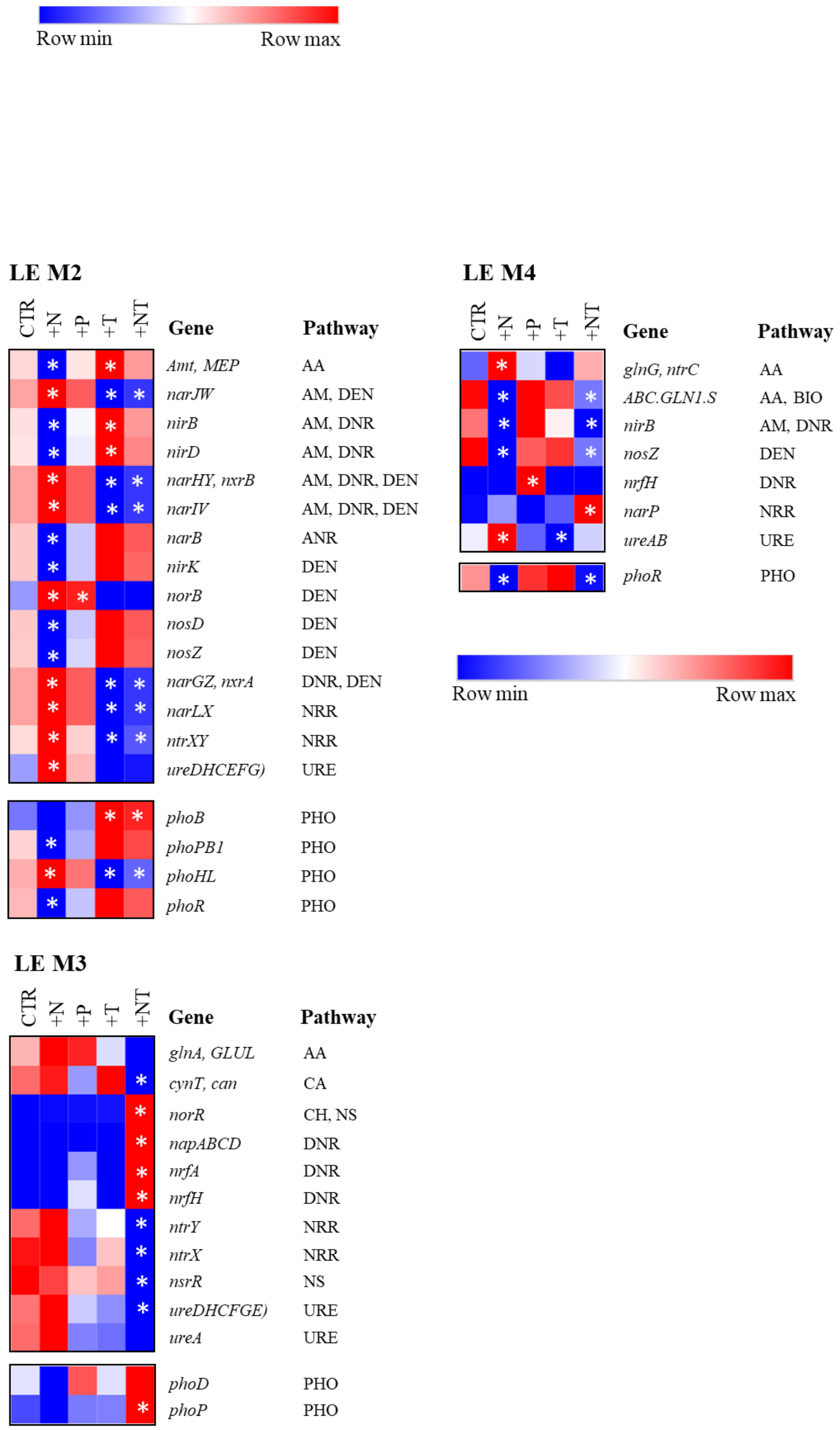

Supplementary Figure 8: Cumulative relative abundance of taxa with *nifH* sequences in NCBI database A) July 24th B) Sept 26th C) M1 D) M2 E) M3 F) M4 experiments. Grey represents taxa classified at the genus level; green represent relative abundance of families with *nifH* containing bacteria to account for sequences that were not classified down to the genus level. P value denotes significance of treatment on the relative abundance of the *nif* containing genera detected with one-way ANOVA and letters above the bars indicate which treatments differ (those that do not share a letter).

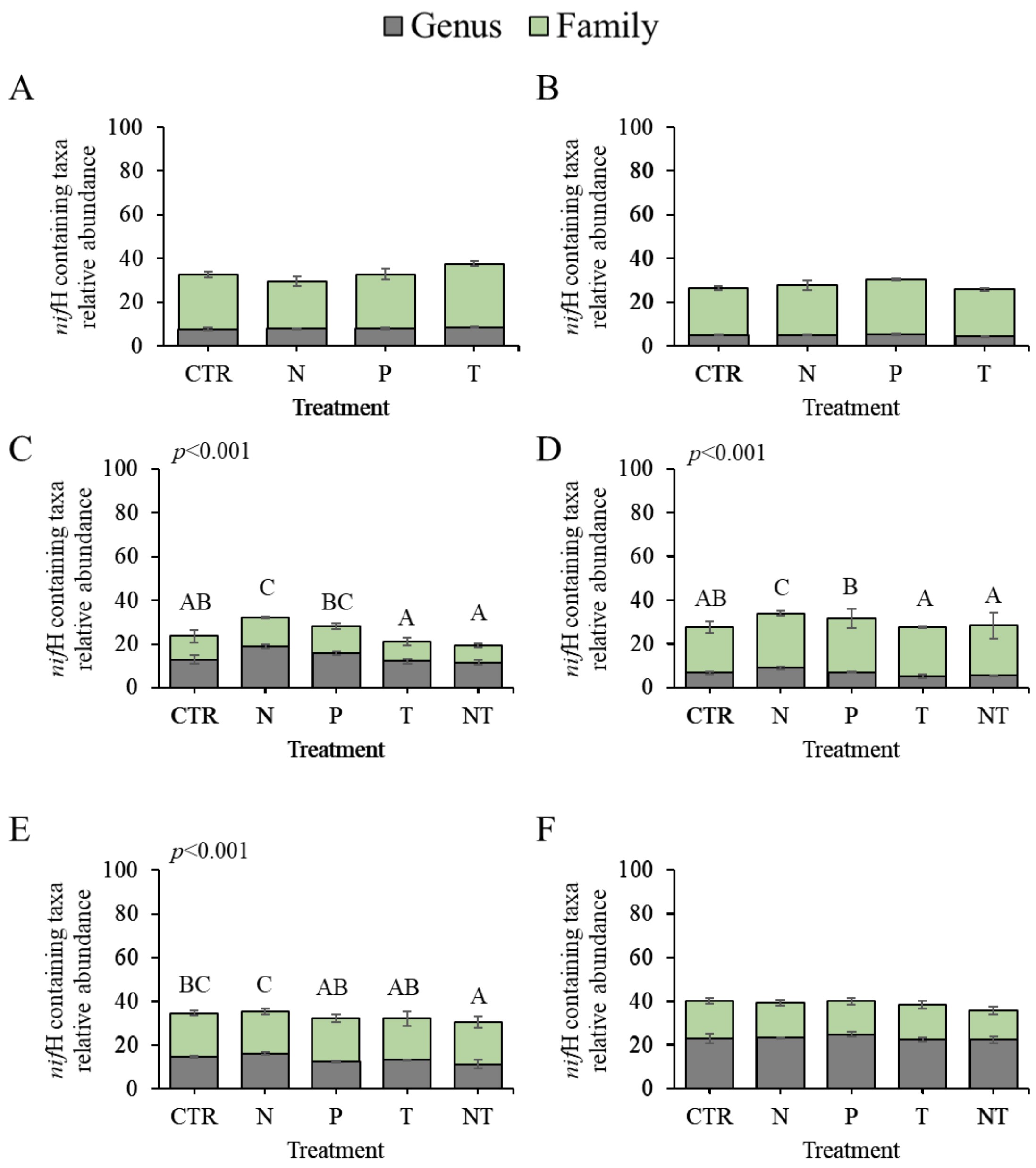

Supplementary Figure 9: Cumulative relative abundance of taxa with *phoX* sequences in NCBI database in the A) July 24th B) Sept 26th C) M1 D) M2 E) M3 F) M4 experiments. Grey represents taxa classified at the genus level, green represent relative abundance of families with *phoX* containing bacteria to account for sequences that were not classified down to the genus level. P value denotes significance of treatment on the relative abundance of the pho containing genera detected with one-way ANOVA and letters above the bars indicate which treatments differ (those that do not share a letter).

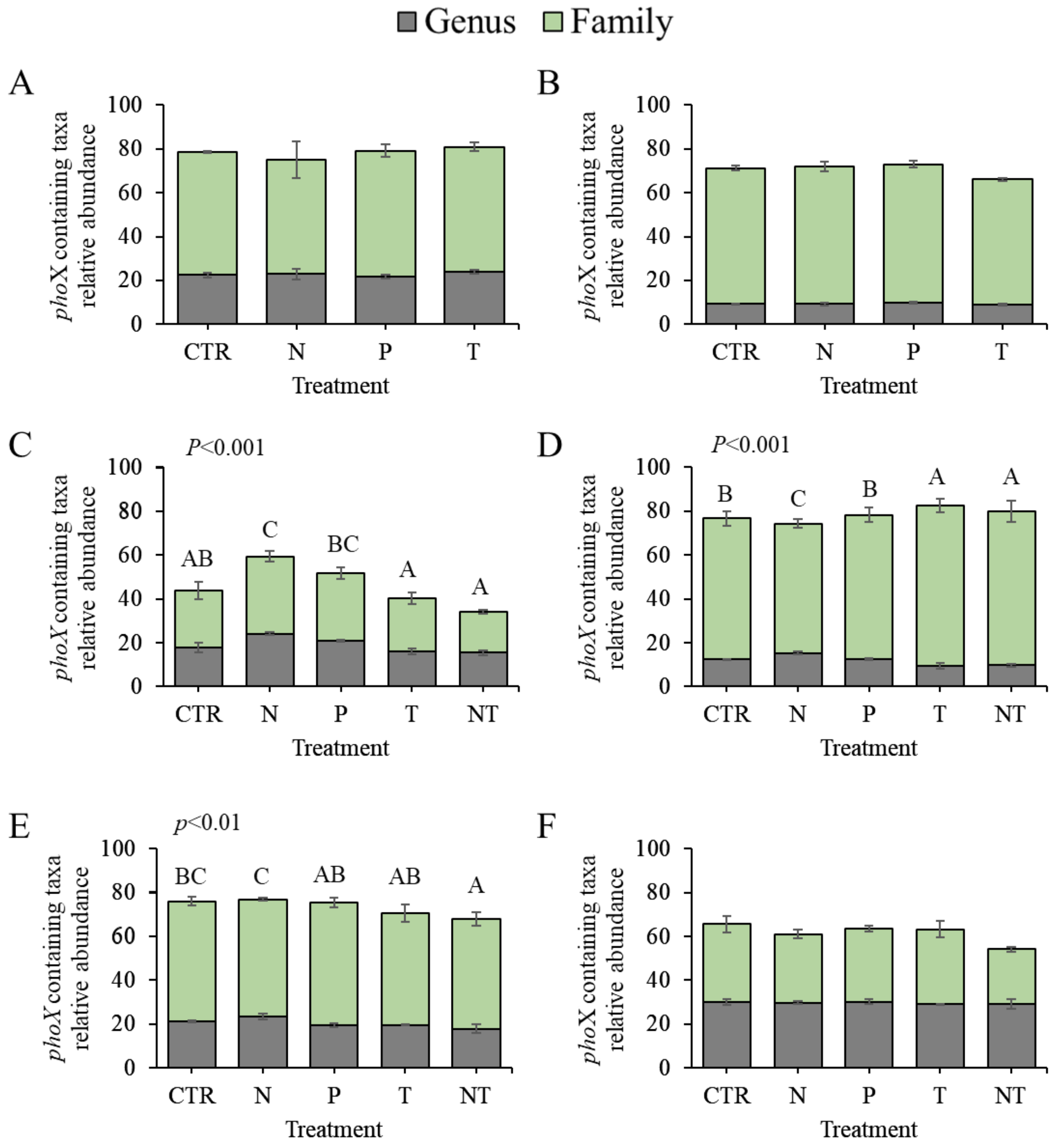

SUPPLEMENTARY TABLES

Supplementary Table 1: Venn diagram analysis results showing the percentage of bacterial taxa shared across the microbiome communities from the initial and final timepoints and among treatments at the final time point. Both the percentage of unique taxa per treatment (only found in one group) and shared across treatments (found in all groups) are shown. The analysis was conducted per experiment at the phylum, class, order, family and ASV taxonomic levels.

		Initial vs final comparison					Final treatment comparison							
**Experiment**	**Taxonomic Level**	**Unique Initial (%)**	**Unique Control (%)**	**Shared (%)**	**Average**	**SD**	**Unique Control (%)**	**Unique N (%)**	**Unique P (%)**	**Unique T (%)**	**Unique NT (%)**	**Shared core (%)**	**Average**	**SD**
July 24th	ASV	48.5	15.5	36	37.67	2.91	6.2	19.8	5.7	12.6		33.4	33.53	3.53
September 26th	ASV	11.1	53	36			11.3	8.0	8.6	7.2		39.5		
M1	ASV	24.7	33	42.2			6.9	5.5	5.3	8.0	6.6	31.2		
M2	ASV	20.2	39.7	40.1			7.3	5.5	4.1	5.5	9.0	34.7		
M3	ASV	23.1	39.6	37.2			2.7	5.5	7.5	9.8	12.5	29.1		
M4	ASV	7	58.6	34.5			7.2	2.8	3.0	9.3	12.7	33.3		
July 24th	Family	50	3.9	46.1	60.73	7.95	4.6	16.8	1.5	8.4		48.9	57.37	6.34
September 26th	Family	10.2	32.2	57.6			12.7	6.3	6.3	4.9		52.8		
M1	Family	21.2	14.4	64.4			1.7	3.4	2.5	4.2	2.5	58.8		
M2	Family	16.3	18.5	65.2			7.9	3.4	0.0	10.1	0.0	67.4		
M3	Family	17.5	14.4	68			1.5	5.2	3.0	3.0	8.2	56.7		
M4	Family	8.1	28.8	63.1			3.8	0.0	0.8	2.3	5.3	59.5		
July 24th	Order	46.8	1.8	51.4	64.63	8.09	3.8	15.2	1.3	6.3		59.5	64.79	4.44
September 26th	Order	12	29.3	58.7			11.9	3.6	6.0	3.6		61.9		
M1	Order	14.7	11.8	73.5			2.8	2.8	2.8	2.8	2.8	63.4		
M2	Order	14.8	18	67.2			8.6	3.4	0.0	3.4	0.0	72.4		
M3	Order	17.7	12.9	69.4			2.7	5.5	7.5	9.8	12.5	66.2		
M4	Order	7.4	25	67.6			4.0	0.0	0.0	2.7	5.3	65.3		
July 24th	Class	52.9	0	47.1	73.28	13.70	7.1	7.1	0.0	7.1	7.1	71.4	76.78	4.44
September 26th	Class	10.7	14.3	75			3.3	0.0	13.3	0.0		73.3		
M1	Class	11.5	3.8	84.6			0.0	3.7	3.7	0.0	3.7	81.5		
M2	Class	12	4	84			4.3	0.0	0.0	0.0	0.0	82.6		
M3	Class	15.4	11.5	73.1			0.0	0.0	4.0	0.0	0.0	76.0		
M4	Class	6.9	17.2	75.9			6.9	0.0	0.0	0.0	0.0	75.9		
July 24th	Phylum	43.3	0	56.7	77.58	12.02	0.0	5.6	0.0	0.0		72.2	77.75	6.01
September 26th	Phylum	6.3	12.5	81.3			0.0	0.0	5.6	0.0		77.8		
M1	Phylum	5.9	0	94.1			0.0	0.0	10.5	0.0	0.0	73.7		
M2	Phylum	11.1	11.1	77.8			0.0	0.0	0.0	0.0	0.0	75.0		
M3	Phylum	5.6	16.7	77.8			0.0	0.0	5.6	0.0	0.0	88.9		
M4	Phylum	5.6	16.7	77.8			10.5	0.0	0.0	0.0	0.0	78.9		

Supplementary Table 2: Significance testing for main effects of treatment on the beta diversity of the bacterial communities using PERMANOVA analysis. Communities were analyzed on a per experiment basis; p value <0.05 used as the threshold for a significant main effect of the treatments on the community structure.

Experiment	Sample size	Variable	Number of groups	pseudo-F	p-value
July 24th	12	Treatment	4	4.47285	0.002
Sept 26th	12	Treatment	4	5.67239	0.001
LEM1	15	Treatment	5	12.094	0.001
LEM2	15	Treatment	5	5.83098	0.001
LEM3	15	Treatment	5	4.98459	0.001
LEM4	15	Treatment	5	4.34664	0.001

Supplementary Table 3: Post hoc analysis of PERMANOVA and PERMDIP on the beta diversity of the ASV inferred bacterial communities between lakes, experiments and treatments at the final timepoints. PERMANOVA analysis was used to examine if the beta diversity significantly differed between groups while PERMDISP was used to test for significant differences in dispersion between groups (i.e. if one group had a more varied response to a variable than another). The lake column indicates what samples were included in the analysis (All: all samples, LA: all samples from Lake Agawam, LE: all samples from Lake Erie, Experiment names: all samples from those experiments). Comparison indicates what variable was tested (grouping by lake, experiment, or treatment) with group indicating which two groups were compared. P values were adjusted for multiple comparisons with an adjusted p-value <0.05 considered significant.

						PERMANOVA			PERMDISP		
Lake	Comparison	Group 1	Group 2	Sample size	Permutations	pseudo-F	p-value	adj. p-value	pseudo-F	p-value	adj. p-value
LA and LE	Lakes	Lake Agawam	Lake Erie	84	999	105.46	0.001	0.001			
LA and LE	Experiments	July 24th	Sept 26th	24	999	65.89	0.001	0.001	7.84	0.001	0.00
LA and LE	Experiments	July 24th	LEM1	27	999	193.24	0.001	0.001	0.62	0.054	0.10
LA and LE	Experiments	July 24th	LEM2	27	999	226.93	0.001	0.001	0.39	0.24	0.33
LA and LE	Experiments	July 24th	LEM3	27	999	196.79	0.001	0.001	0.00	1	1.00
LA and LE	Experiments	July 24th	LEM4	27	999	209.56	0.001	0.001	0.06	0.876	0.94
LA and LE	Experiments	Sept 26th	LEM1	27	999	278.62	0.001	0.001	19.54	0.001	0.00
LA and LE	Experiments	Sept 26th	LEM2	27	999	360.22	0.001	0.001	15.34	0.001	0.00
LA and LE	Experiments	Sept 26th	LEM3	27	999	303.47	0.001	0.001	9.75	0.001	0.00
LA and LE	Experiments	Sept 26th	LEM4	27	999	322.51	0.001	0.001	21.20	0.001	0.00
LA and LE	Experiments	LEM1	LEM2	30	999	136.99	0.001	0.001	3.97	0.001	0.00
LA and LE	Experiments	LEM1	LEM3	30	999	74.16	0.001	0.001	0.80	0.1	0.17
LA and LE	Experiments	LEM1	LEM4	30	999	32.82	0.001	0.001	0.60	0.243	0.33
LA and LE	Experiments	LEM2	LEM3	30	999	36.98	0.001	0.001	0.57	0.314	0.39
LA and LE	Experiments	LEM2	LEM4	30	999	137.57	0.001	0.001	2.11	0.001	0.00
LA and LE	Experiments	LEM3	LEM4	30	999	61.64	0.001	0.001	0.10	0.455	0.53
LA and LE	Treatments	Control	N	36	999	0.18	0.95	0.981			
LA and LE	Treatments	Control	NT	30	999	2.78	0.05	0.197			
LA and LE	Treatments	Control	P	36	999	0.11	0.981	0.981			
LA and LE	Treatments	Control	T	36	999	0.32	0.851	0.981			
LA and LE	Treatments	N	NT	30	999	2.96	0.043	0.197			
LA and LE	Treatments	N	P	36	999	0.25	0.902	0.981			
LA and LE	Treatments	N	T	36	999	0.53	0.669	0.981			
LA and LE	Treatments	NT	P	30	999	2.74	0.059	0.197			
LA and LE	Treatments	NT	T	30	999	2.52	0.088	0.220			
LA and LE	Treatments	P	T	36	999	0.35	0.86	0.981			
LA	Treatments	Control	N	12	999	0.15	0.794	0.794			
LA	Treatments	Control	P	12	999	0.24	0.577	0.730			
LA	Treatments	Control	T	12	999	1.26	0.213	0.608			
LA	Treatments	N	P	12	999	0.26	0.608	0.730			
LA	Treatments	N	T	12	999	1.16	0.275	0.608			
LA	Treatments	P	T	12	999	1.12	0.304	0.608			
LE	Treatments	Control	N	24	999	0.51	0.632	0.790			
LE	Treatments	Control	NT	24	999	0.91	0.394	0.788			
LE	Treatments	Control	P	24	999	0.27	0.871	0.871			
LE	Treatments	Control	T	24	999	0.51	0.61	0.790			
LE	Treatments	N	NT	24	999	1.74	0.172	0.788			
LE	Treatments	N	P	24	999	0.88	0.36	0.788			
LE	Treatments	N	T	24	999	1.37	0.236	0.788			
LE	Treatments	NT	P	24	999	0.94	0.375	0.788			
LE	Treatments	NT	T	24	999	0.39	0.734	0.816			
LE	Treatments	P	T	24	999	0.64	0.524	0.790			
July 24th	Treatments	Control	N	6	999	1.65	0.113	0.170			
July 24th	Treatments	Control	P	6	999	1.54	0.198	0.238			
July 24th	Treatments	Control	T	6	999	16.23	0.094	0.170			
July 24th	Treatments	N	P	6	999	1.29	0.309	0.309			
July 24th	Treatments	N	T	6	999	5.41	0.097	0.170			
July 24th	Treatments	P	T	6	999	10.55	0.098	0.170			
Sept 26th	Treatments	Control	N	6	999	1.17	0.214	0.214			
Sept 26th	Treatments	Control	P	6	999	2.85	0.093	0.176			
Sept 26th	Treatments	Control	T	6	999	11.86	0.105	0.176			
Sept 26th	Treatments	N	P	6	999	2.01	0.202	0.214			
Sept 26th	Treatments	N	T	6	999	11.44	0.117	0.176			
Sept 26th	Treatments	P	T	6	999	7.30	0.115	0.176			
LEM1	Treatments	Control	N	6	999	14.42	0.102	0.116			
LEM1	Treatments	Control	NT	6	999	7.86	0.096	0.116			
LEM1	Treatments	Control	P	6	999	3.37	0.1	0.116			
LEM1	Treatments	Control	T	6	999	3.71	0.087	0.116			
LEM1	Treatments	N	NT	6	999	35.42	0.108	0.116			
LEM1	Treatments	N	P	6	999	8.60	0.103	0.116			
LEM1	Treatments	N	T	6	999	24.79	0.1	0.116			
LEM1	Treatments	NT	P	6	999	16.01	0.101	0.116			
LEM1	Treatments	NT	T	6	999	4.94	0.116	0.116			
LEM1	Treatments	P	T	6	999	8.57	0.109	0.116			
LEM2	Treatments	Control	N	6	999	5.29	0.095	0.135			
LEM2	Treatments	Control	NT	6	999	5.81	0.1	0.135			
LEM2	Treatments	Control	P	6	999	1.96	0.192	0.213			
LEM2	Treatments	Control	T	6	999	7.52	0.097	0.135			
LEM2	Treatments	N	NT	6	999	9.63	0.104	0.135			
LEM2	Treatments	N	P	6	999	4.75	0.1	0.135			
LEM2	Treatments	N	T	6	999	16.17	0.105	0.135			
LEM2	Treatments	NT	P	6	999	4.25	0.108	0.135			
LEM2	Treatments	NT	T	6	999	0.97	0.507	0.507			
LEM2	Treatments	P	T	6	999	5.75	0.091	0.135			
LEM3	Treatments	Control	N	6	999	1.61	0.097	0.116			
LEM3	Treatments	Control	NT	6	999	7.44	0.094	0.116			
LEM3	Treatments	Control	P	6	999	2.65	0.088	0.116			
LEM3	Treatments	Control	T	6	999	4.06	0.082	0.116			
LEM3	Treatments	N	NT	6	999	7.91	0.097	0.116			
LEM3	Treatments	N	P	6	999	3.68	0.091	0.116			
LEM3	Treatments	N	T	6	999	4.96	0.116	0.116			
LEM3	Treatments	NT	P	6	999	5.63	0.089	0.116			
LEM3	Treatments	NT	T	6	999	4.90	0.102	0.116			
LEM3	Treatments	P	T	6	999	2.98	0.109	0.116			
LEM4	Treatments	Control	N	6	999	2.39	0.092	0.117			
LEM4	Treatments	Control	NT	6	999	5.36	0.1	0.117			
LEM4	Treatments	Control	P	6	999	2.73	0.099	0.117			
LEM4	Treatments	Control	T	6	999	1.92	0.208	0.208			
LEM4	Treatments	N	NT	6	999	5.57	0.094	0.117			
LEM4	Treatments	N	P	6	999	6.53	0.105	0.117			
LEM4	Treatments	N	T	6	999	4.62	0.105	0.117			
LEM4	Treatments	NT	P	6	999	7.73	0.088	0.117			
LEM4	Treatments	NT	T	6	999	4.72	0.094	0.117			
LEM4	Treatments	P	T	6	999	4.95	0.095	0.117			

Supplementary Table 4: Alpha diversity metrics of the ASV-derived bacterial communities per sample. Pielou's evenness, the number of observed ASV's per sample and the Shannon index are shown.

Experiment	Treatment	Replicate	Pielou e	Observed ASVs	Shannon index
July 24th	CTR	1	0.730	173	5.43
July 24th	CTR	2	0.727	189	5.50
July 24th	CTR	3	0.743	183	5.59
July 24th	N	1	0.735	193	5.58
July 24th	N	2	0.736	157	5.37
July 24th	N	3	0.772	239	6.10
July 24th	P	1	0.754	196	5.74
July 24th	P	2	0.737	176	5.50
July 24th	P	3	0.715	162	5.25
July 24th	T	1	0.709	181	5.32
July 24th	T	2	0.734	161	5.38
July 24th	T	3	0.716	235	5.64
Sept 26th	CTR	1	0.664	241	5.25
Sept 26th	CTR	2	0.671	247	5.34
Sept 26th	CTR	3	0.660	240	5.22
Sept 26th	N	1	0.661	227	5.17
Sept 26th	N	2	0.664	228	5.20
Sept 26th	N	3	0.656	209	5.06
Sept 26th	P	1	0.652	225	5.10
Sept 26th	P	2	0.675	231	5.30
Sept 26th	P	3	0.676	229	5.30
Sept 26th	T	1	0.688	220	5.35
Sept 26th	T	2	0.679	218	5.27
Sept 26th	T	3	0.700	186	5.28
LEM1	CTR	1	0.552	221	4.30
LEM1	CTR	2	0.609	226	4.76
LEM1	CTR	3	0.612	238	4.83
LEM1	N	1	0.693	223	5.41
LEM1	N	2	0.704	203	5.39
LEM1	N	3	0.685	214	5.31
LEM1	P	1	0.640	187	4.83
LEM1	P	2	0.639	180	4.79
LEM1	P	3	0.651	216	5.05
LEM1	T	1	0.579	255	4.63
LEM1	T	2	0.616	211	4.76
LEM1	T	3	0.601	229	4.71
LEM1	NT	1	0.543	212	4.20
LEM1	NT	2	0.549	216	4.26
LEM1	NT	3	0.560	270	4.53
LEM2	CTR	1	0.618	154	4.49
LEM2	CTR	2	0.623	155	4.53
LEM2	CTR	3	0.615	156	4.48
LEM2	N	1	0.683	135	4.83
LEM2	N	2	0.665	147	4.79
LEM2	N	3	0.666	162	4.89
LEM2	P	1	0.627	150	4.53
LEM2	P	2	0.629	146	4.52
LEM2	P	3	0.632	135	4.47
LEM2	T	1	0.556	131	3.91
LEM2	T	2	0.599	137	4.25
LEM2	T	3	0.603	152	4.37
LEM2	NT	1	0.610	150	4.41
LEM2	NT	2	0.613	138	4.36
LEM2	NT	3	0.627	144	4.50
LEM3	CTR	1	0.718	213	5.55
LEM3	CTR	2	0.713	207	5.48
LEM3	CTR	3	0.722	179	5.41
LEM3	N	1	0.711	228	5.57
LEM3	N	2	0.722	205	5.54
LEM3	N	3	0.691	299	5.68
LEM3	P	1	0.683	270	5.52
LEM3	P	2	0.700	268	5.65
LEM3	P	3	0.684	286	5.59
LEM3	T	1	0.708	314	5.87
LEM3	T	2	0.711	327	5.94
LEM3	T	3	0.680	265	5.48
LEM3	NT	1	0.704	293	5.77
LEM3	NT	2	0.720	298	5.92
LEM3	NT	3	0.752	332	6.30
LEM4	CTR	1	0.764	297	6.28
LEM4	CTR	2	0.746	287	6.09
LEM4	CTR	3	0.746	272	6.03
LEM4	N	1	0.748	269	6.04
LEM4	N	2	0.748	248	5.95
LEM4	N	3	0.749	255	5.99
LEM4	P	1	0.746	249	5.94
LEM4	P	2	0.748	235	5.89
LEM4	P	3	0.746	246	5.93
LEM4	T	1	0.744	291	6.09
LEM4	T	2	0.745	310	6.17
LEM4	T	3	0.733	326	6.12
LEM4	NT	1	0.721	322	6.01
LEM4	NT	2	0.731	350	6.18
LEM4	NT	3	0.739	351	6.25

Supplementary Table 5: Average percent dissimilarity of the bacterial community beta diversity due to treatment calculated using SIMPER analysis. Percent dissimilarity was calculated on both the ASV derived bacterial communities and the PICRUST predicted metagenomic content of the ASV derived bacterial communities. Dissimilarities were calculated on a per experimental basis with Columns Group 1 and Group 2 indicating which treatments were compared. Larger percent dissimilarities indicate greater variation between the beta diversities between the groups.

			ASV infered	PICRUST infered
Experiment	Group1	Group2	Mean dissimilarity (%)	Mean dissimilarity (%)
July 24th	CTR	N	20.26	6.01
July 24th	CTR	P	13.93	2.301
July 24th	CTR	T	30.62	4.922
July 24th	N	P	20	5.95
July 24th	N	T	37.73	6.448
July 24th	P	T	33.09	4.759
Sept 26th	CTR	N	9.593	1.906
Sept 26th	CTR	P	11.18	3.358
Sept 26th	CTR	T	18.65	3.859
Sept 26th	N	P	11.09	2.303
Sept 26th	N	T	17.84	4.197
Sept 26th	P	T	16.97	4.341
LEM1	CTR	N	29.03	17.19
LEM1	CTR	P	22.97	9.504
LEM1	CTR	T	19.99	5.08
LEM1	CTR	NT	21.13	12.27
LEM1	N	P	24.27	10.38
LEM1	N	T	32.81	18.22
LEM1	N	NT	37.08	27.03
LEM1	P	T	25.44	11.16
LEM1	P	NT	32.19	20.12
LEM1	T	NT	19.75	10.8
LEM2	CTR	N	20.33	6.56
LEM2	CTR	P	16.06	4.471
LEM2	CTR	T	20.5	6.691
LEM2	CTR	NT	22.96	5.149
LEM2	N	P	19.3	5.573
LEM2	N	T	28.68	10.95
LEM2	N	NT	24.53	8.888
LEM2	P	T	20.88	7.062
LEM2	P	NT	20.8	5.498
LEM2	T	NT	16.55	4.209
LEM3	CTR	N	21.24	3.7
LEM3	CTR	P	38.48	4.854
LEM3	CTR	T	36.83	5.667
LEM3	CTR	NT	40.13	12.81
LEM3	N	P	29.23	4.374
LEM3	N	T	30.03	4.811
LEM3	N	NT	36.25	11.46
LEM3	P	T	18.25	3.233
LEM3	P	NT	26.57	9.167
LEM3	T	NT	24.94	8.337
LEM4	CTR	N	21.41	6.894
LEM4	CTR	P	24.89	6.199
LEM4	CTR	T	22.75	6.447
LEM4	CTR	NT	26.98	13.63
LEM4	N	P	19.61	7.504
LEM4	N	T	22.92	7.842
LEM4	N	NT	23.94	12.35
LEM4	P	T	27.55	6.509
LEM4	P	NT	27.02	15.11
LEM4	T	NT	24.28	12.63
All	July 24th	Sept 26t	54.59	
All	July 24th	LEM1	88.62	
All	July 24th	LEM2	83.93	
All	July 24th	LEM3	83.54	
All	July 24th	LEM4	87.25	
All	Septemb	LEM1	90.87	
All	Septemb	LEM2	86.81	
All	Septemb	LEM3	87.48	
All	Septemb	LEM4	89.53	
All	LEM1	LEM2	63.8	
All	LEM1	LEM3	54.39	
All	LEM1	LEM4	39.87	
All	LEM2	LEM3	39.11	
All	LEM2	LEM4	60.96	
All	LEM3	LEM4	48.06	
All	Lake Aga	Lake Eri	87.25	

Supplementary Table 6: Post hoc analysis of PERMANOVA and PERMDIP on the beta diversity of the PICRUST predicted metagenomes of the bacterial communities between lakes, experiments and treatments at the final timepoints. PERMANOVA analysis was used to examine if the beta diversity significantly differed between groups while PERMDISP was used to test for significant differences in dispersion between groups (i.e. if one group had a more varied response to a variable than another). The selection column indicates what samples were included in the analysis (All: all samples, LA: all samples from Lake Agawam, LE: all samples from Lake Erie, Experiment names: all samples from those experiments). Comparison indicates what variable was tested (grouping by lake, experiment, or treatment) with group indicating which two groups were compared. P values were adjusted for multiple comparisons with an adjusted p-value <0.05 considered significant.'

						PERMANOVA				PERMDISP		
Selection	Comparison	Group 1	Group 2	Sample size	Permutations	pseudo-F	p-value	adj p-value	n effect pv	pseudo-F	p-value	adj p-value
LAandLE	Lake	LakeAgawam	LakeErie	84	999	43.646	0.001	0.001	0.001			
LAandLE	Experiment	July 24th	Sept 26th	24	999	31.760	0.001	0.001	0.001	3.045	0.040	0.055
LAandLE	Experiment	July 24th	LEM1	27	999	143.261	0.001	0.001	0.001	9.047	0.001	0.003
LAandLE	Experiment	July 24th	LEM2	27	999	166.923	0.001	0.001	0.001	0.143	0.574	0.662
LAandLE	Experiment	July 24th	LEM3	27	999	116.122	0.001	0.001	0.001	0.101	0.621	0.665
LAandLE	Experiment	July 24th	LEM4	27	999	174.568	0.001	0.001	0.001	3.872	0.002	0.004
LAandLE	Experiment	Sept 26th	LEM1	27	999	141.452	0.001	0.001	0.001	17.504	0.001	0.003
LAandLE	Experiment	Sept 26th	LEM2	27	999	178.731	0.001	0.001	0.001	15.292	0.001	0.003
LAandLE	Experiment	Sept 26th	LEM3	27	999	136.363	0.001	0.001	0.001	5.461	0.001	0.003
LAandLE	Experiment	Sept 26th	LEM4	27	999	194.249	0.001	0.001	0.001	13.347	0.001	0.003
LAandLE	Experiment	LEM1	LEM2	30	999	143.172	0.001	0.001	0.001	11.218	0.001	0.003
LAandLE	Experiment	LEM1	LEM3	30	999	98.771	0.001	0.001	0.001	9.518	0.002	0.004
LAandLE	Experiment	LEM1	LEM4	30	999	13.257	0.001	0.001	0.001	2.372	0.128	0.160
LAandLE	Experiment	LEM2	LEM3	30	999	24.786	0.001	0.001	0.001	0.002	0.957	0.957
LAandLE	Experiment	LEM2	LEM4	30	999	169.909	0.001	0.001	0.001	4.435	0.006	0.010
LAandLE	Experiment	LEM3	LEM4	30	999	90.885	0.001	0.001	0.001	3.428	0.028	0.042
LAandLE	Treatment	Control	N	36	999	0.329	0.748	0.943	0.074			
LAandLE	Treatment	Control	NT	30	999	3.572	0.046	0.153	0.074			
LAandLE	Treatment	Control	P	36	999	0.102	0.943	0.943	0.074			
LAandLE	Treatment	Control	T	36	999	0.135	0.910	0.943	0.074			
LAandLE	Treatment	N	NT	30	999	6.010	0.006	0.060	0.074			
LAandLE	Treatment	N	P	36	999	0.384	0.682	0.943	0.074			
LAandLE	Treatment	N	T	36	999	0.727	0.481	0.943	0.074			
LAandLE	Treatment	NT	P	30	999	4.883	0.015	0.075	0.074			
LAandLE	Treatment	NT	T	30	999	2.958	0.081	0.202	0.074			
LAandLE	Treatment	P	T	36	999	0.305	0.770	0.943	0.074			
July 24th	Treatment	Control	N	6	999	0.389	0.709	0.910	0.651			
July 24th	Treatment	Control	P	6	999	0.613	0.788	0.910	0.651			
July 24th	Treatment	Control	T	6	999	6.415	0.107	0.321	0.651			
July 24th	Treatment	N	P	6	999	0.218	0.910	0.910	0.651			
July 24th	Treatment	N	T	6	999	0.420	0.693	0.910	0.651			
July 24th	Treatment	P	T	6	999	3.328	0.098	0.321	0.651			
Sept 26th	Treatment	Control	N	6	999	4.160	0.104	0.104	0.001			
Sept 26th	Treatment	Control	P	6	999	13.956	0.087	0.104	0.001			
Sept 26th	Treatment	Control	T	6	999	19.483	0.100	0.104	0.001			
Sept 26th	Treatment	N	P	6	999	2.206	0.094	0.104	0.001			
Sept 26th	Treatment	N	T	6	999	12.274	0.098	0.104	0.001			
Sept 26th	Treatment	P	T	6	999	14.758	0.090	0.104	0.001			
LEM1	Treatment	Control	N	6	999	30.789	0.107	0.129	0.001			
LEM1	Treatment	Control	NT	6	999	10.845	0.100	0.129	0.001			
LEM1	Treatment	Control	P	6	999	10.115	0.101	0.129	0.001			
LEM1	Treatment	Control	T	6	999	0.737	0.505	0.505	0.001			
LEM1	Treatment	N	NT	6	999	48.393	0.096	0.129	0.001			
LEM1	Treatment	N	P	6	999	9.728	0.098	0.129	0.001			
LEM1	Treatment	N	T	6	999	28.576	0.116	0.129	0.001			
LEM1	Treatment	NT	P	6	999	30.013	0.092	0.129	0.001			
LEM1	Treatment	NT	T	6	999	7.424	0.098	0.129	0.001			
LEM1	Treatment	P	T	6	999	11.109	0.100	0.129	0.001			
LEM2	Treatment	Control	N	6	999	5.119	0.101	0.246	0.003			
LEM2	Treatment	Control	NT	6	999	1.279	0.404	0.417	0.003			
LEM2	Treatment	Control	P	6	999	0.710	0.398	0.417	0.003			
LEM2	Treatment	Control	T	6	999	3.083	0.187	0.312	0.003			
LEM2	Treatment	N	NT	6	999	21.446	0.123	0.246	0.003			
LEM2	Treatment	N	P	6	999	6.343	0.099	0.246	0.003			
LEM2	Treatment	N	T	6	999	25.359	0.110	0.246	0.003			
LEM2	Treatment	NT	P	6	999	2.726	0.238	0.340	0.003			
LEM2	Treatment	NT	T	6	999	1.000	0.417	0.417	0.003			
LEM2	Treatment	P	T	6	999	4.595	0.112	0.246	0.003			
LEM3	Treatment	Control	N	6	999	2.595	0.211	0.234	0.002			
LEM3	Treatment	Control	NT	6	999	22.358	0.095	0.173	0.002			
LEM3	Treatment	Control	P	6	999	9.567	0.100	0.173	0.002			
LEM3	Treatment	Control	T	6	999	7.343	0.098	0.173	0.002			
LEM3	Treatment	N	NT	6	999	14.629	0.104	0.173	0.002			
LEM3	Treatment	N	P	6	999	4.768	0.122	0.174	0.002			
LEM3	Treatment	N	T	6	999	2.974	0.191	0.234	0.002			
LEM3	Treatment	NT	P	6	999	10.658	0.083	0.173	0.002			
LEM3	Treatment	NT	T	6	999	6.148	0.093	0.173	0.002			
LEM3	Treatment	P	T	6	999	0.812	0.640	0.640	0.002			
LEM4	Treatment	Control	N	6	999	1.201	0.495	0.535	0.001			
LEM4	Treatment	Control	NT	6	999	12.106	0.093	0.151	0.001			
LEM4	Treatment	Control	P	6	999	1.016	0.431	0.535	0.001			
LEM4	Treatment	Control	T	6	999	0.766	0.535	0.535	0.001			
LEM4	Treatment	N	NT	6	999	21.031	0.099	0.151	0.001			
LEM4	Treatment	N	P	6	999	8.323	0.096	0.151	0.001			
LEM4	Treatment	N	T	6	999	5.171	0.099	0.151	0.001			
LEM4	Treatment	NT	P	6	999	46.205	0.106	0.151	0.001			
LEM4	Treatment	NT	T	6	999	18.755	0.094	0.151	0.001			
LEM4	Treatment	P	T	6	999	4.674	0.099	0.151	0.001			
